# Resistance loci affecting distinct stages of fungal pathogenesis: use of introgression lines for QTL mapping and characterization in the maize - *Setosphaeria turcica *pathosystem

**DOI:** 10.1186/1471-2229-10-103

**Published:** 2010-06-08

**Authors:** Chia-Lin Chung, Joy M Longfellow, Ellie K Walsh, Zura Kerdieh, George Van Esbroeck, Peter Balint-Kurti, Rebecca J Nelson

**Affiliations:** 1 Dept. of Plant Pathology and Plant-Microbe Biology, Cornell University, Ithaca, NY 14853, USA; 2 Dept. of Plant Breeding and Genetics, Cornell University, Ithaca, NY 14853, USA; 3 Dept. of Biology, West Virginia State University, Institute, WV 25112, USA; 4 Dept. of Crop Science, North Carolina State University, Raleigh, NC 27695, USA; 5 USDA-ARS, Plant Science Research Unit; Dept. of Plant Pathology, North Carolina State University, Raleigh, NC 27695, USA

## Abstract

**Background:**

Studies on host-pathogen interactions in a range of pathosystems have revealed an array of mechanisms by which plants reduce the efficiency of pathogenesis. While R-gene mediated resistance confers highly effective defense responses against pathogen invasion, quantitative resistance is associated with intermediate levels of resistance that reduces disease progress. To test the hypothesis that specific loci affect distinct stages of fungal pathogenesis, a set of maize introgression lines was used for mapping and characterization of quantitative trait loci (QTL) conditioning resistance to *Setosphaeria turcica*, the causal agent of northern leaf blight (NLB). To better understand the nature of quantitative resistance, the identified QTL were further tested for three secondary hypotheses: (1) that disease QTL differ by host developmental stage; (2) that their performance changes across environments; and (3) that they condition broad-spectrum resistance.

**Results:**

Among a set of 82 introgression lines, seven lines were confirmed as more resistant or susceptible than B73. Two NLB QTL were validated in BC_4_F_2 _segregating populations and advanced introgression lines. These loci, designated *qNLB1.02 *and *qNLB1.06*, were investigated in detail by comparing the introgression lines with B73 for a series of macroscopic and microscopic disease components targeting different stages of NLB development. Repeated greenhouse and field trials revealed that *qNLB1.06_Tx303 _*(the Tx303 allele at bin 1.06) reduces the efficiency of fungal penetration, while *qNLB1.02_B73 _*(the B73 allele at bin 1.02) enhances the accumulation of callose and phenolics surrounding infection sites, reduces hyphal growth into the vascular bundle and impairs the subsequent necrotrophic colonization in the leaves. The QTL were equally effective in both juvenile and adult plants; *qNLB1.06_Tx303 _*showed greater effectiveness in the field than in the greenhouse. In addition to NLB resistance, *qNLB1.02_B73 _*was associated with resistance to Stewart's wilt and common rust, while *qNLB1.06_Tx303 _*conferred resistance to Stewart's wilt. The non-specific resistance may be attributed to pleiotropy or linkage.

**Conclusions:**

Our research has led to successful identification of two reliably-expressed QTL that can potentially be utilized to protect maize from *S. turcica *in different environments. This approach to identifying and dissecting quantitative resistance in plants will facilitate the application of quantitative resistance in crop protection.

## Background

Pathogenesis is the series of events that occurs in a host-pathogen interaction, including infection and colonization of the host, and reproduction and dissemination of the pathogen. Genetic variation in host and/or pathogen can have quantitative or qualitative effects on the extent of disease. Many plant genetic factors that modulate pathogenesis have been discovered. The best known group is the R-genes, which provide high levels of resistance or even complete immunity. R-gene mediated resistance is initiated through a gene-for-gene interaction; the recognition of a pathogen effector by a host protein encoded by the R-gene leads to the induction of the hypersensitive response (HR), the production of antimicrobial metabolites such as phytoalexins, and the expression of pathogenesis-related (PR) proteins [1]. This type of interaction, typically resulting in a highly effective but race-specific defense response against pathogenic invasion, is sometimes known as qualitative resistance. Quantitative resistance, on the other hand, confers intermediate levels of resistance and is believed to be controlled by a set of genes distinct from, or partially overlapping with, those involved in qualitative resistance [2-7].

Although each quantitative resistance locus conditions a relatively small effect on pathogenesis, this type of resistance is of agricultural interest because qualitative resistance tends to be ephemeral in many pathosystems and is unavailable in others. Quantitative resistance is presumably more durable because multiple genes with minor effects lead to lower selection pressure and greater complexity to overcome [8]. A large number of quantitative trait loci (QTL) for disease resistance have been mapped in plants [6,9], but little is known about the underlying genetic basis or defense mechanisms involved. A range of genetic mechanisms controlling basal resistance, defense signalling pathways, detoxification, morphology, and development in the plant host, is hypothesized to be associated with reducing disease progress [6]. A small number of quantitative resistance genes have recently been cloned [3-5,7,10], implicating diverse host functions in quantitative resistance.

Given that diverse host functions affect quantitative resistance, it is likely that QTL act at different stages of pathogenesis. The ways in which quantitative resistance affects different stages of pathogenesis has been addressed, to a limited extent, by comparing trait values obtained using distinct (usually macroscopic) disease components. In most (or probably all) of the phytopathosystems analyzed to date, differences in various disease parameters can be observed among plant genotypes. Previous QTL studies for foliar diseases have mapped distinct loci associated with incubation period, lesion number, lesion size, or diseased leaf area, with results suggesting that defense genes affecting lesion formation and lesion expansion may not be the same. In breeding programs, selection for decreased lesion length or lesion numbers can have insignificant effects on incubation period or disease severity (eg. [11]). These observations suggest that distinct resistance mechanisms govern different macroscopic components of resistance.

More insights into the role of a given disease QTL in limiting pathogenesis can be gained through histopathological analysis. While biochemical and microscopic analyses have been applied to investigate major gene resistance and fungal pathogenicity factors (reviewed by Vidhyasekaran [12]), few studies have reported the effect of individual QTL on distinct stages of pathogenesis from a microscopic view (exceptions include [5,13]). If QTL effective at specific stages of pathogenesis can be identified, combining favorable alleles for complementary QTL (eg. for infection and colonization) will likely provide greater levels of resistance.

Northern leaf blight (NLB; also known as turcicum blight and northern corn leaf blight) of maize was used as a model system to identify and characterize disease QTL at the macroscopic and microscopic levels. NLB, caused by *Setosphaeria turcica *(anamorph *Exserohilum turcicum*, syn. *Helminthosporium turcicum*), is one of the most prevalent foliar diseases in most maize-growing regions of the world. The disease causes periodic epidemics associated with significant yield losses [14-17], particularly under conditions of moderate temperature and high humidity [18]. Qualitative and quantitative forms of resistance against *S. turcica *are available in maize germplasm [19,20], and have been widely utilized alone or in combination in resistance breeding programs [21]. A few histological studies have revealed the pathogenesis of *S. turcica *on maize leaves by staining, whole mount and serial dissection [22-25]. Marked phenotypic variation in symptom development has been observed among diverse maize lines in our multiple field and greenhouse trials. How macroscopic and microscopic phenotypes relate to specific QTL remains to be determined.

To answer questions concerning individual QTL effects, such as testing the hypothesis that distinct QTL act at different stages of pathogenesis, well-defined genetic stocks that differ only at specific loci are required. Introgression lines have been successfully used to study QTL in maize [26,27], rice [28], barley [29,30], tomato [31], and Arabidopsis [32]. While QTL analysis using recombinant inbred lines (RILs) provides greater statistical power in detecting QTL [33], RIL-based approaches have limitations in estimating QTL effects [31,32,34]. Introgression lines can be efficiently used to produce near-isogenic lines (NILs), which permit careful analysis of phenotypic effects associated with introgressed segments [27,31].

NILs allow many long-standing questions about quantitative disease resistance to be addressed, such as the relationship between disease QTL and plant maturity, the interaction of QTL and environmental factors, and the specificity of resistance conditioned by QTL. The interplay between disease resistance and plant development has been widely recognized [35] yet remains poorly understood. In general, the resistance in adult plants or older leaves is greater than in juvenile plants or younger leaves (eg. [36-38]), and a correlation between resistance and flowering time has been found [[39], R. Wisser, J. Kolkman, and P. Balint-Kurti, unpublished]. Some QTL effects may thus be specific to certain plant developmental stages. In addition, the expression and effectiveness of many genes/QTL have been observed to be regulated by environmental conditions [9,40,41]. Another issue of fundamental and practical interest is whether a disease QTL confers specific or broad-spectrum resistance. A single locus can condition resistance to more than one disease, if it encompasses linked QTL effective against different diseases, or its underlying genes are involved in broad-spectrum resistance pathways.

Here, we describe the use of introgression lines and derived NILs for QTL mapping and macro-/microscopic characterization in the maize - *S. turcica *pathosystem. We used an available population of introgression lines named TBBC3 [27]. This population is composed of introgression lines, each of which carries one or a few chromosomal segments of the donor genotype Tx303 in the genetic background of the recurrent parent B73. To better understand the nature of quantitative resistance, we assembled a panel of conventional and novel disease components targeting different stages of disease development. These were used to demonstrate that two QTL affect different stages of pathogenesis. The QTL were further characterized to shed light on three secondary hypotheses: (1) that disease QTL differ by host developmental stage (young versus adult plants); (2) that their performance changes across environments (field versus greenhouse); and (3) that they condition broad-spectrum resistance. This approach to identifying and dissecting quantitative resistance in plants will facilitate more effective and efficient application of quantitative resistance in crop protection.

## Methods

### Plant materials

A set of 82 TBBC3 introgression lines was provided by J. Holland of the USDA-ARS unit at North Carolina State University. The TBBC3 (for Tx303 by B73 Backcross 3) population, originally developed by C. Stuber at North Carolina State University [26], was the most extensively developed set of introgression lines available at the time for public use in maize. The population was derived from an initial cross of Tx303 and B73, followed by backcrossing to B73 for three generations. Each line was then selfed for several generations to attain homozygosity. Genotypic information was publicly available for each line, consisting of 14 restriction fragment length polymorphism (RFLP) and 116 simple sequence repeat (SSR) markers across the genome. Each line was known to carry one or more Tx303 introgressions, covering on average 2.5% of the genome, in the background of the sequenced reference maize line B73. Taken together, the set of introgression lines collectively carries ~89% of Tx303 genome [27].

To validate and characterize the effects of Tx303 introgressions, several BC_4_F_2 _populations were developed by crossing selected TBBC3 lines to B73. Sets of BC_4_F_3 _and BC_4_F_4 _lines carrying different introgression(s) were subsequently derived by single-seed descent. After four generations of marker-assisted backcrossing, the BC_4_F_3 _and BC_4_F_4 _lines were designated as NILs.

### Assessments of northern leaf blight

A single isolate of *S. turcica *(NY001, race 1) was used in the experiments carried out in New York (NY), and a mixture of isolates representing race 1, race 23, and race 23N of *S. turcica *was used in North Carolina (NC). For preparation of liquid inoculum, *S. turcica *was cultured for two to three weeks on lactose - casein hydrolysate agar (LCA) plates under a 12 hr/12 hr normal light-dark cycle at room temperature. The conidia were then dislodged with sterile ddH_2_O and a glass rod, filtered through four layers of cheesecloth, and adjusted to the final concentration with the aid of a haemocytometer. Solid inoculum was prepared by culturing *S. turcica *on sorghum grains in plastic milk jugs for two to three weeks under the same condition. For each jug, 900 ml of sorghum grains were soaked overnight in 600 ml of water in a milk jug. The jug was then autoclaved twice at 121°C, 15 lb/cm^2^, for 25 minutes per run. The jugs were inoculated by dividing the spore suspension produced from one heavily colonized LCA plate (10 cm diameter) among five jugs. Jugs were shaken daily to prevent caking and accelerate fungal colonization.

In this study, the "juvenile" phase refers to earlier vegetative stages, and the "adult" phase refers to later vegetative stages and reproductive stages. Juvenile plants (at the five- to six-leaf stage) and adult plants (late vegetative stage, around two weeks before tasselling) were used for inoculation. The inoculation technique utilized depended on the specific objectives of the experiment. In the field trials in NY, plants were inoculated with both liquid (0.5 ml of spore suspension, 4 × 10^3 ^conidia per ml, 0.02% Tween 20) and solid inoculum (1/4 teaspoon, ~1.25 ml of colonized sorghum grains) placed in the whorl. This was done to ensure the viability of inoculum across a range of conditions (under optimal conditions, the liquid inoculum was considered most effective, while the solid inoculum was considered to perform more effectively under dry conditions). In the field trials in NC, ~20 grains of sorghum colonized with *S. turcica *were placed in the whorl.

In greenhouse trials, whorl inoculation with aforementioned liquid inoculum was carried out for assessing individual plants in the segregating populations. Spray inoculation was performed for detailed QTL characterization using NILs, as it provides significantly better differentiation for NLB evaluation (data not shown). The spray method was preferred for microscopic examination and real-time PCR quantification. A higher number of spores could be evenly distributed on leaf surface with spraying, making the subsequent sampling more effective and accurate. About 0.5 ml of concentrated spore suspension (5 × 10^4 ^conidia per ml, 0.02% Tween 20) was evenly sprayed on the first fully expanded leaf with an airbrush (Badger^® ^Model 150) at 20 psi. After inoculation, the plants were kept overnight in a mist chamber at > 85% RH, then maintained at 22°C day/18°C night temperature with a 14 hr-light/10 hr-dark cycle.

### Phenotypic characterization of resistance to NLB

Field experiments were conducted at Cornell's Robert Musgrave Research Farm in Aurora, NY and Central Crops Field Station in Clayton, NC. Plants were evaluated for different disease parameters and for days to anthesis (DTA). DTA, which was only assessed for field-grown plants, was scored on a row basis when > 50% of the plants in a row started to shed pollen. An overview of various disease components used in this study and their corresponding stages during NLB development is summarized in Table 1. The evaluation method for each parameter is illustrated as below.

**Table 1:** Overview of disease components used to target different stages of northern leaf blight (NLB) development.

Disease component	Description (unit)	Targeted disease development stage(s)	Evaluation	Literature
Incidence of multiple appressoria^a^	The incidence of > 1 appressorium developed from each germinated conidium (%)	Pre-penetration	Trypan blue staining and microscopy	[25,42]
Infection efficiency	The incidence of successful infection per germinated conidium (%)	Penetration into the epidermal cell	Trypan blue staining and microscopy, KOH-aniline blue fluorescence microscopy	[25,42,94]
Accumulation of callose and phenolics^a^	Diameter of enhanced fluorescing area surrounding the infection site (μm)	Intercellular and intracellular hyphal growth from primary infected cell to surrounding mesophyll cells	KOH-aniline blue fluorescence microscopy	[43]
Vascular invasion efficiency^a^	The incidence of hyphae entering vascular bundles per infection site (%)	Hyphal growth into the vasculature	KOH-aniline blue fluorescence microscopy	[43]
Fungal biomass ratio^a^	The percentage of fungal DNA divided by the total DNA in the infected leaf tissues (%)	Overall fungal growth in leaves before the appearance of necrotic lesions	DNA-based real-time quantitative PCR	[44]
Incubation period (IP)	The number of days from inoculation to the appearance of the first lesion on a plant (days)	Xylem plugging due to extensive hyphal growth in the vascular veins	Visual examination	[73,74,76-78,95]
Lesion expansion (LE)	The longitudinal expansion of a lesion per day (mm)	Destructive hyphal growth in primary inoculated leaves	Digital caliper measurement	[78,96]
Diseased leaf area (DLA)	The percentage of infected leaf area of the entire plant, disregarding decayed bottom leaves (%)	Destructive hyphal growth on the leaves of a entire plant, caused by primary and secondary inoculum	Visual examination	[73,76,77,94,97]
Disease severity^b^	The severity of infected leaf area of the entire plant (scale 1-10, 1: little diseased area)	Destructive hyphal growth on the leaves of a entire plant, caused by primary and secondary inoculum	Visual examination	[98]
Area under the disease progress curve (AUDPC)	Total area under the graph of DLA or other disease severity rating (area unit)	An overall destructive hyphal growth on a plant throughout the season	Calculated from visual examination scores	[76,77]

^a ^To authors' knowledge, not previously utilized as a disease component for evaluating NLB resistance.

^b ^Only applied in the 2006 trial in North Carolina.

#### Microscopic analysis

Two microscopy techniques were applied to investigate differential development of *S. turcica *in the NILs: trypan blue staining and KOH-aniline blue staining. In greenhouse trials, infected leaf samples were harvested from individual plants. In the field trial, for the purpose of obtaining a sufficient number of infection sites for examination, samples (per genotype per block) were collected from four plants in a row and pooled for subsequent treatments.

Trypan blue staining was performed as previously described [25,42] with some modifications. Infected tissues were collected at two days post inoculation (dpi) from plants in the greenhouse, and at 3 dpi from plants in the field trials. Leaf samples were cut into 1 × 1 cm^2 ^segments, cleared first in an acetic acid: ethanol (1:3, v/v) solution overnight, then in an acetic acid: ethanol: glycerol (1:5:1, v/v/v) solution for at least 3 hours. The samples were subsequently incubated overnight in a staining solution of 0.01% (w/v) trypan blue in lactophenol, and rinsed then stored in 60% glycerol until examination. Specimens were transferred onto microscopic slides and examined under a compound microscope. Fifty to 60 germinated conidia were assessed per individual plant (greenhouse) or per row (field).

A modified KOH-aniline blue fluorescence technique [43] was used to visualize the growth of fungal hyphae inside the infected leaves and the accumulation of (plant-produced) callose and phenotypic compounds around the infection sites. Infected leaves were sampled at 4 dpi and 7 dpi in greenhouse trials, and at 6 dpi in the field trial. The samples were cut into 1 × 1 cm^2 ^segments, incubated in 1 M KOH at room temperature for 24 hours, then autoclaved at 121°C, 15 lb/cm^2 ^for 2-5 min. Autoclaving time was adjusted according to the rigidity of leaves, which varied with plant genotype and maturity. The autoclaved specimens were rinsed in ddH_2_O three times, then stored in autoclaved ddH_2_O until examination. Specimens were carefully placed on microscopic slides and mounted in a staining solution of 0.05% aniline blue in 0.067 M K_2_HPO_4 _(prepared at least 2 hrs prior to use). Thirty five to 40 germinated conidia were checked per individual plant (greenhouse) or per row (field) under a Zeiss fluorescence microscope with a G365 excitation filter, a FT395 dichromatic beam splitter, and an LP420 barrier filter.

#### Quantitative real-time PCR for quantifying fungal colonization

DNA-based quantitative PCR (qPCR) was performed as described by Qi and Yang [44] with some modifications. The specific pair of primers for *S. turcica*: forward: 5'-TCTTTTGCGCACTTGTTGTT and reverse: 5'-CGATGCCAGAACCAAGAGAT, was designed based on the internal transcribed spacer 1 (ITS1) of ribosomal DNA gene in *S. turcica*. The ITS1 sequence (GenBank: AF163067.1) was obtained from the nucleotide database of the National Center for Biotechnology Information (NCBI). PCR amplification resulted in a specific fragment of 170 base pairs.

Inoculation experiments were performed three times in the greenhouse. Five plants per genotype were spray-inoculated. The same amounts of infected tissue (0.12 g per plant) were collected at 9 dpi from the middle part of each leaf. Leaf samples were ground with liquid nitrogen and DNA was extracted following the protocol described later. The extracted DNA from each individual plant was dissolved in 100 μl TE buffer. Total DNA concentration was determined using the PicoGreen^® ^dsDNA quantitation assay kit (Invitrogen, Eugene, Oregon, USA). Fungal DNA was quantified using qPCR. The ratio of fungal biomass in maize leaves was computed from the amount of fungal DNA divided by total DNA.

Each qPCR reaction was performed in a total volume of 25 μl, containing 12.5 μl of iTaq SYBR^® ^Green Supermix with ROX (Bio-Rad Laboratories, Hercules, CA, USA), 3 μl of 7.5-fold diluted DNA from an infected plant and 300 nM of each forward and reverse primer. PCR samples were incubated in an ABI Prism 7000 Sequence Detection System (Applied Biosystems, Foster City, CA, USA) with thermal cycling parameters of 95°C for 2 min followed by 40 cycles of 15 sec at 95°C and 30 sec at 56°C. Two standard curves were constructed by mixing a series of *S. turcica *DNA (0, 1, 10, 50, 100, 500 and 1000 pg) with 50 ng of maize DNA extracted from non-inoculated B73 and Tx303 plants, respectively. The quantification of fungal biomass in infected B73 and derived NILs was based on the first standard curve (created from mixing with a constant amount of B73 DNA), while the quantification for DNA from infected Tx303 was based on the second standard curve. Three technical replicates were carried out in individual plates for both Picogreen quantification and iTaq SYBR Green PCR, with the samples for standard curves repeated twice in the same plates.

#### Incubation period (IP)

Individual plants were checked every day after 7 dpi for the appearance of the first wilted lesions. The number of dpi when the first lesions were observed was scored as the IP. In the trials at Aurora NY, IP scores were rated for individual plants, then averaged for the rows. In the trial at Clayton NC, IP was recorded on a row basis when > 50% of the plants in a row started showing lesions.

#### Lesion expansion (LE)

LE was scored in the trials in NY but not NC. Around two to three weeks after inoculation, three lesions per plant were randomly chosen for measurement. Lesion margins were marked and then measured 10-14 days later for the longitudinal extension with a digital caliper. The expansion measurements taken from three lesions were averaged, and divided by the number of days from the marking until measurement of the lesions.

#### Primary diseased leaf area (PrimDLA)

Primary DLA was rated as the percentage of infected leaf area of the inoculated leaves in the 2006 trial in NY. It was scored once on a row basis at around three to four weeks after inoculation.

#### Diseased leaf area (DLA)

DLA was rated as the percentage of infected leaf area of the entire plant, disregarding decayed bottom leaves. DLA was rated on a row basis for TBBC3 lines and derived NILs, and on individual plants for testing trait-marker association in segregating populations. DLA was rated three to four times per season at 10-14 day intervals. The first DLA score was recorded at one to two weeks after observing the onset of secondary infection.

#### Disease severity

Disease severity was rated on a row basis four times through the season in the 2006 trial in NC. The severity score was based on a 1 to 9 scale corresponding to the percentage of infected leaf area on primarily the ear leaf as well as the leaves above and below the ear leaf (severity 1: 0%, 2: 12.5%, 3: 25%, ...., 9: 100%).

#### Area under the disease progress curve (AUDPC)

The AUDPC was calculated as , where *y_i _*= DLA or disease severity at time *i*, *t_i+1 _*- *t_i _*= day interval between two ratings, *n *= number of ratings [45].

### Evaluation for multiple disease resistance

#### Stewart's wilt

*Pantoea stewartii *(syn. *Erwinia stewartii*) strain PsNY003, originally collected in NY in 1991, was obtained from Helene Dillard of Cornell University. Inoculum was prepared as previously described [46] with the use of nutrient agar plates and nutrient broth as media. Plants at the five- to six-leaf stage were inoculated with *P. stewartii *following the pinprick method [47,48] with a modified inoculator. The multiple-pin inoculator was made with 30 T-pins (1.5 inch long), pieces of 5.5 cm × 6.5 cm sponge and cork board (3/8 inch thick) fastened on two arms of a tong with rubber bands. Primary diseased leaf area was rated as the percentage of infected area of the inoculated leaves. It was scored twice (two and three weeks after inoculation) on a row basis and the scores were averaged.

#### Anthracnose stalk rot

A New York isolate of *Colletotrichum graminicola *(teleomorph: *Glomerella graminicola*) (isolate Cg151) was obtained from Gary Bergstrom of Cornell University. Each plant was inoculated with 1 ml of 10^6 ^conidia per ml (0.02% Tween 20) when more than 50% of the plants in every row were tasseling [49]. Inoculum preparation and inoculation were conducted as described by Muimba-Kankolongo and Bergstrom [50], with the replacement of plastic straw with 1 ml pipette tips. Four weeks after inoculation, stalks were split longitudinally and the percentages of discolored area of individual internodes were visually rated [49] and summed for analysis. In 2007, eight consecutive internodes were scored from four plants per row (inoculation was conducted in NLB plot); in 2008, six consecutive internodes were scored from eight plants per row.

#### Common smut

In the 2007 trial, plants in NLB plots were evaluated for the development of ear galls and stalk galls resulting from natural infection. Artificial inoculation was conducted in the 2008 trial using six compatible strains of *Ustilago maydis *(UmNY001, UmNY002, UmNY003, UmNY004, UmNY008 and UmNY009), which were isolated from naturally infected smut galls collected at Aurora NY in 2007. *U. maydis *strains were cultured as described by du Toit and Pataky [51]. Inoculum was prepared by adjusting sporidial suspension (in potato dextrose broth) to a final concentration of 10^6 ^sporidia per ml (0.02% Tween 20) with sterilized distilled water. Equal amounts of sporidia from the six compatible strains were mixed prior to inoculation. Non-pollinated (shoot-bagged) primary ear of each plant was injected with 2 ml of mixed sporidial suspension at the time that the silks of most primary ears had emerged 1-5 cm. Every plant in the row was rated from four weeks after inoculation for ear galls on a 0-10 scale, corresponding to the number and size of galls and the disease severity of the entire plant (0 = no smut galls and 10 = a dead smut-infected plant). Stalk galls resulting from natural infection were also scored.

#### Common rust

Urediniospores of *Puccinia sorghi *were collected from naturally infected leaves at Aurora NY in 2007. Inoculum was increased on three- to four-leaf stage seedlings of susceptible sweet corn in the greenhouse. About 300 mg of stock urediniospores (preserved at -80°C) were suspended in 100 ml of Sortrol oil (Chevron Phillips Chemical Company, Phillips, TX, USA) [52] and evenly applied on leaves with a spray gun (Preval, Yonkers, NY, USA). Plants were kept overnight in a mist chamber at > 85% RH, then grown for two more weeks until *Puccinia sorghi *sporulated vigorously. The urediniospores were collected by agitating infected leaves with mature rust pustules in distilled water and filtering through four layers of cheesecloth. Spore suspension was adjusted to a final concentration of 2 × 10^5 ^urediniospores per ml with the aid of a haemocytometer. Field plants were inoculated at the six- to eight-leaf stage by adding 1 ml of spore suspension (0.02% Tween 20) in the whorl [53]. Evaluation for disease severity was based on a 0-10 scale with 0.5 increments, corresponding to the percentage of infected leaf area of the entire plant (0 = no disease, 1 = 10%, ..., 10 = 100%). Disease severity was scored three times at 9-day intervals from four weeks after inoculation in 2008, and at 15-20 day intervals from 10 days after inoculation in 2009. AUDPC was calculated from the three severity scores as described above.

### DNA extraction and genotyping

About 0.1 g of fresh or lyophilized leaf tissue and a stainless steel ball (5/32 inch diameter, OPS Diagnostics, NJ, USA) were loaded in each well of a 96-well plate (Corning^® ^Costar 96 Well Polypropylene Cluster Tubes). Tissues were frozen at -80°C and pulverized at 450 strokes/min for 50-120 sec using Genogrinder 2000 (SPEX CertiPrep Inc., Metuchen, NJ, USA). Genomic DNA was extracted following a standard CTAB extraction protocol [54,55] with 500 μl of CTAB extraction buffer, 400 μl of chlorophorm/isoamyl alcohol (24:1, v/v), and 300 μl of isopropanol. Fungal DNA was extracted as described, except that *S. turcica *mycelium was ground in liquid nitrogen with a pestle and mortar, and transferred to a 1.5 ml eppendorf tube for later steps.

Simple sequence repeat (SSR) markers were used for genotypic analysis following a single-reaction nested PCR method [56]. Each PCR reaction was performed as described by Wisser *et al*. [57] in a total volume of 13 μl, with the same thermal cycling parameters as described by Schuelke [56]. Amplicons labeled with different fluorescent dyes were multiplexed (combining up to four PCR reactions, 0.7 μl PCR product per specific primer pair), mixed with 9 μl formamide and 0.05-0.1 μl GeneScan-500 LIZ size standard (Applied Biosystems), and analyzed on the Applied BioSystems 3730xl DNA Analyzer at Biotechnology Resource Center at Cornell University. The sizes of amplicons were scored using GeneMapper v. 3.0 (Applied Biosystems).

### Experimental design

The full set of 82 TBBC3 lines, a subset of 15 TBBC3 lines, and derived sets of BC_4_F_3 _and BC_4_F_4 _NILs were evaluated in the field following the resolvable incomplete block design (also known as an alpha design [58]). The 82 TBBC3 lines were evaluated at Aurora NY for IP, PrimDLA, DLA, and DTA. To precisely estimate the effects of Tx303 introgressions, every experimental row was grown next to a row of B73 (B73 in every third row). The whole design had two replicates with 14 blocks per replicate, and six experimental rows plus three B73 rows per block. A parallel experiment was conducted on the 82 TBBC3 lines at Clayton NC with two replicates, nine blocks per replicate and 10 rows per block. The B73 rows were included in every other block for the control (five B73 rows per replicate). Disease severity and DTA were evaluated. The field trials on the 15 selected TBBC3 lines were conducted at Aurora NY and Clayton NC following the alpha design with B73 in every third row as described above. The 15 lines were selected from those significantly different from B73 in NLB resistance (based on the first-year results), and/or from the lines carrying Tx303 introgression(s) corresponding to previously identified NLB QTL. In each location, there were four replicates with four blocks per replicate, and four experimental rows plus two B73 rows per block. The IP, DLA, and DTA were evaluated. An additional disease component, lesion expansion (LE), was evaluated at Aurora NY as well. During the same field season, to understand the effectiveness of identified QTL in adult plants, the same 15 selected TBBC3 lines were grown in a separate field plot at Aurora NY, following identical arrangement and design with three replicates. The plants were inoculated two weeks before flowering (rather than the usual five- to six-leaf stage), and evaluated for IP and DLA.

To associate resistance with specific introgressions, several BC_4_F_2 _populations were genotyped and phenotyped for IP, LE, and DLA in either greenhouse or in the field at Aurora NY. Individual plants of each population were grown within a single block with B73 rows as the border. For QTL confirmation, derived BC_4_F_3 _and BC_4_F_4 _NILs were evaluated at Aurora NY for IP, LE, DLA, and DTA, following the previously described alpha design with B73 rows in every third row. The experiment was planted in two replicates, with five blocks per replicate, and four experimental rows plus two B73 rows per block.

To test whether the identified NLB QTL confer resistance to other important maize diseases, derived BC_4_F_3 _and BC_4_F_4 _NILs were evaluated at Aurora NY for anthracnose stalk rot (ASR) and common smut in 2007 and 2008, and for Stewart's wilt and common rust in 2008 and 2009. In 2007, the assessments of artificially inoculated ASR and naturally occurring common smut were conducted on the plants in NLB trial. In 2008 and 2009, derived NILs were grown in separate field plots for different disease evaluations. Trials for Stewart's wilt and common rust were planted following the previously described alpha design, with two replicates, five blocks per replicate, and four experimental rows plus two B73 rows per block. Trials for ASR were planted following the same alpha design, with two replicates, two blocks per replicate, and seven experimental rows plus four B73 rows per block. Plants in the trials of common smut were randomized in two replicates.

Four genotypes were used for microscopic analysis and DNA-based real time PCR quantification: B73, Tx303, TBBC3-38-05F (the NIL with *qNLB1.06_Tx303_*), and TBBC3-42-10E-02 (the NIL with *qNLB1.02_Tx303_*). Three greenhouse trials (each with five plants per genotype) and one field trial (five blocks, one row per genotype per block) were conducted from December 2007 to July 2008. Real-time PCR quantification was not performed in the 2008 field trial. In the greenhouse, plants subject to the same treatment were randomized within a block in order to eliminate the variance due to environmental factors.

### Data analysis

Mixed model analyses were performed in JMP 7.0 for the field trails following an alpha design. For trials conducted in one field location, "maize lines" was specified as a fixed factor, whereas "replicates" and "blocks within replicates" were specified as random. Data from the 2006 trials in NY and NC were analyzed separately because of the different field arrangements and disease rating scales. A combined analysis across two locations (NY and NC) was conducted for the 2007 trials by fitting a mixed model with "maize lines", "locations" and "maize lines by locations" as fixed factors, and "replicates within locations" and "blocks within replicates within locations" as random effects. Phenotypic differences between TBBC3 lines/NILs and B73, or between any two NILs, were determined by pair-wise comparisons of least squares means using two-tailed Student's t-test at *P *< 0.05. Correction for multiple comparisons was not made since all the tests were independent. The TBBC3 lines and derived NILs were each compared to the recurrent parent B73, and pairs of NILs were only compared to each other when sharing identical genotypes except for the target introgression(s) under testing. Overall comparisons of TBBC3 lines or NILs were not intended in this analysis.

The QTL effect of each marker locus was investigated in the full TBBC3 population. Considering the presence of unlinked, potentially unrecognized introgressed segments in the same lines, a series of statistical tests was conducted as described by Szalma *et al*. [27] with some modification. Markers likely associated with resistance (*P *< 0.05) were identified by: 1) comparing the least squares mean of TBBC3 lines homozygous for Tx303 alleles at each locus to the least squares mean of B73 rows, and 2) performing one-way analysis of variance (ANOVA) on an individual marker - trait basis in the TBBC3 population. Significant markers were grouped into linked introgression blocks, and the markers with the greatest significance in each linked block were considered to reflect the most likely QTL position. Correlations between the most significant markers were checked. For each pair of unlinked but correlated markers, TBBC3 lines fixed for one marker were grouped and analyzed for the co-segregation of the second marker and disease traits, using two-tailed Student's t test at *P *< 0.05. A putative QTL was declared if the mean of all the lines with Tx303 alleles at this locus was significantly different from B73, and significant difference was detected for lines contrasting for this locus under the condition of another potential QTL locus being fixed.

In segregating populations, the phenotypic and genotypic data were first analyzed by mixed stepwise regression, with a significance probability of *P *< 0.05 for each parameter to enter/leave the model [59]. Once the markers significantly associated with the traits were identified, ANOVA was conducted on a single marker basis at *P *< 0.05 to estimate QTL effects. For the populations with more than one marker showing significant effect on traits, the effective markers were further tested for epistatic interactions by examining marker pairs by two-way ANOVA. Allele effects were determined by pair-wise two-tailed Student's t test according to the least significant difference (LSD) at *P *= 0.05.

The proportion data from microscopic analysis and qPCR were transformed to arcsine of the square roots prior to statistical analysis. Arcsine transformed data were analyzed by fitting a linear least squares model with "genotype" and "replicate" as two independent variables, and with the number in the denominator of each proportion as a weighting factor. For qPCR, considering the biological and technical replicates, the fungal biomass ratios were also arcsine square-root transformed, but were analyzed with a different linear least squares model of y_ijm _= β_0 _+ β_1 _(genotype_i_) + β_2 _(rep_j_) + β_3 _(plate_m _(rep_j_)) + e_ijm _(genotype i = B73, Tx303 and two derived NILs; rep j = 1, 2, 3; plate m = 1, 2, 3; e_ijm_: random experimental error). Least squares means and 95% confidence intervals were back-transformed to percentages. Confidence intervals were larger than significance levels due to asymmetry resulting from back transformation of arcsine scale. The significant QTL effects were estimated by pair-wise comparisons of least squares means of introgression lines/NILs and B73 using two-tailed Student's t-test at *P *< 0.05. All the statistical analyses in the study were conducted using JMP 7.0.

## Results

**Figure 1:**
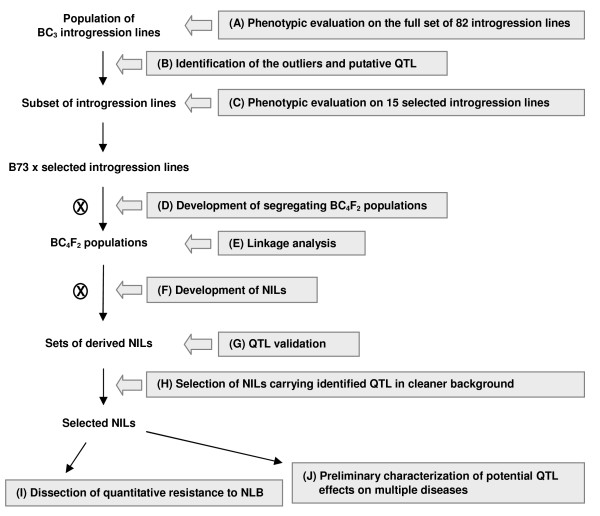
**Strategy for mapping and characterizing QTL using introgression lines**. (A) Initial screening was conducted at Aurora NY and Clayton NC. Conventional disease components, including incubation period (IP), diseased leaf area (DLA), and disease severity were evaluated. (B) Lines that differed from B73, and lines showed extreme phenotypes in the population were determined. Putative QTL were also identified. (C) Lines carrying putative NLB QTL were evaluated to confirm their differential resistance/susceptibility relative to B73. Plants were tested at the juvenile and adult stages for IP, LE, and DLA. (D) Selected lines were backcrossed to B73, then selfed to generate F_2 _populations segregating for the introgressed regions within the populations. (E) Individual plants were tested for markers targeting introgressed regions, and co-segregation was assessed with the disease components measured in the greenhouse or field in NY. Candidate NLB QTL were determined if trait-marker association was detected in more than one F_2 _population. (F) The BC_3_F_3 _and/or BC_3_F_4 _near-isogenic lines (NILs) carrying different Tx303 introgressions were derived from F_2 _populations. (G) The effects of different introgressions on conventional disease components were further tested. The NLB QTL were declared if the QTL effects were validated in the NILs. (H) Selected NILs carrying identified QTL were used for detailed QTL characterization. (I) Selected NILs were evaluated with a series of disease components targeting different stages of disease development (Table 1) in the greenhouse and field. (J) Selected NILs were also evaluated for anthracnose stalk rot, common rust, common smut and Stewart's wilt at Aurora NY.

A stepwise strategy (Figure 1) was conducted to map and characterize QTL using the TBBC3 introgression lines and derived NILs.

### Identification of outliers and putative NLB QTL in the TBBC3 population

The full set of 82 TBBC3 lines was screened for NLB resistance at field sites in New York and North Carolina in 2006. As shown in Additional file 1, the TBBC3 population was generally more susceptible than B73 at both locations. In the trial at Aurora NY, three lines were scored as significantly more resistant and 20 lines were scored as significantly more susceptible than B73 for primary DLA, while no lines showed significantly more resistance and 39 lines showed more susceptibility than B73 based on AUDPC. In the trial at Clayton NC, eight lines were significantly more resistant and 13 lines were significantly more susceptible than B73 for AUDPC. The only line that showed contrasting phenotypes in the two environments was TBBC3-25, suggesting experimental error or environmental influence on the expression of NLB QTL.

A combination of analytic approaches was used to associate the resistance or susceptibility effects with specific chromosomal segments [27]. Based on this analysis, 13 putative NLB QTL were identified in the TBBC3 population (Additional file 2). Except for bins 1.01 and 5.08-5.09, the putative QTL co-localized with NLB QTL previously detected in four maize mapping populations [20].

### Phenotypic evaluation on the subset of 15 TBBC3 lines

A subset of 15 TBBC3 lines was selected for further evaluation based on the degree of difference in NLB resistance between each individual TBBC3 line and B73 at the two field sites (Additional file 1 and Table 2). Lines significantly or marginally different (*P *< 0.15) from B73 in either of the two locations were taken into consideration. Line selection was also influenced by the identity of the Tx303 introgressions carried by the lines; those carrying introgressions corresponding both to putative QTL identified in the TBBC3 population (Additional file 2), and to previously reported NLB QTL (specifically those at bins 1.07-1.08, 5.02, and 6.04-6.05 [20]) were given priority because of our interest in QTL validation and characterization rather than in QTL discovery.

**Table 2:** NLB resistance of the subset of 15 TBBC3 introgression lines.

Maize line	Introgressed region (Bin)^a^	Days to anthesis	Trait (juvenile plants being inoculated; NY and NC)^b^			Trait (adult plants being inoculated; NY)	
				
			Relative IP	Relative LE	Relative AUDPC	Relative IP	Relatvie AvgDLA
**Tx303**		8.7***	2.1***	-0.3	-164.1***	7.5***	-8.7***
**TBBC3-38**	1.03, 1.06, 5.00, 5.03, 5.07-5.09	4.0***	2.1***	-0.1	-110.2***	2.6***	-4.5***
**TBBC3-39**	1.03, 1.06, 5.00, 5.07	2.4***	0.9*	0.0	-67.2**	1.3*	-4.3***
TBBC3-18	1.11, 7.05, 7.06, 10.03, 10.04	4.2***	0.8*	-0.1	-35.8	1.3*	-2.0**
TBBC3-19^c^	7.04, 7.06, 9.03, 10.04	1.4*	1.2**	0.1	-29.0	1.2	3.2***
TBBC3-26	7.06	1.0	1.4**	0.0	-1.8	-0.1	1.0
TBBC3-75^d^	6.04-6.05	1.2	0.4	0.1	12.2	2.6***	-2.3**
TBBC3-02	1.07-1.08, 5.01-5.02, 8.03-8.05	3.2***	-0.7	0.2	35.7	0.6	1.5*
TBBC3-14^c^	1.09-1.10, 4.07	3.1***	-0.9*	0.0	67.0**	0.3	6.7***
TBBC3-03	1.01, 1.04, 7.03, 8.03-8.05	2.8***	-0.1	-0.1	117.0***	-1.4*	5.7***
TBBC3-61^c^	1.10	1.4*	-0.5	0.6***	126.7***	0.2	7.0***
**TBBC3-77**	1.01-1.03, 6.04-6.05	4.3***	-1.4**	0.9***	139.4***	-1.4*	4.0***
**TBBC3-30**	4.02-4.03, 5.04	0.2	-0.9*	0.6***	195.4***	-0.6	4.7***
**TBBC3-21**	4,07, 9.01-9.04	0.6	-1.7***	0.8***	275.5***	-1.1	7.8***
**TBBC3-36**	1.01, 1.03-1.05, 4.01, 8.08	1.3	-0.8	0.7***	305.6***	-1.8**	8.2***
**TBBC3-42**	1.01-1.02, 4.07-4.08, 5.01-5.02, 8.02-8.05	2.2**	-2.1***	0.7**	310.8***	-1.3*	8.7***

In 2007, the same set of plant materials was inoculated at the five- to six-leaf stage in New York (NY) and North Carolina (NC), and were grown in a separate field plot in NY and inoculated 1-2 weeks before anthesis. Trait values shown are the least squares means of maize lines relative to the recurrent parent B73. Phenotypes that were significantly more resistant than B73 are highlighted in bold, while the phenotypes significantly more susceptible than B73 are underscored. The significance level was determined by pair-wise two-tailed Student's t test on the least square difference (denoted as * 0.01 <*P *< 0.05; ** 0.001 <*P *< 0.01; *** *P *< 0.001). Maize lines in bold are the ones that were consistently more resistant or more susceptible than B73 across two field environments.

^a ^Introgressed genome regions from Tx303 (heterozygous regions not shown). Bin position was based on genetic map of intermated B73 × Mo17 population (version IBM 2008 neighbors).

^b ^Relative least squares means of incubation period (IP) and area under the disease progress curve (AUDPC) shown were from the combined analysis across the trials in NY and NC. Lesion expansion (LE) was only scored in NY.

^c ^Individual TBBC3 lines in each field environment were inspected. TBBC3-19 was significantly more resistant than B73 for IP (*P *= 0.03), DLA1 (*P *= 0.001), DLA2 (*P *= 0.035) and AUDPC (*P *= 0.010) only in NC. TBBC3-14 and TBBC3-61 showed greater susceptibility than B73 for three DLA ratings (*P *< 0.003) and AUDPC (*P *< 0.0001) only in NY. The same differential degrees of resistance or susceptibility in two field environments were also observed in 2006 (Additional file 1).

^d ^Resistance to NLB was more effective in adult plants.

In 2007, the 15 selected lines were evaluated at the same field locations in NY and NC to confirm their disease phenotypes. Significant "line" effects (*P *< 0.0001) were found for all disease parameters in each of NY and NC, and across two locations. In the combined analysis across environments, significant effects of "location" (*P *< 0.002 for IP, DLA and AUDPC) and "line-by-location" (*P *< 0.0001 for DLA and AUDPC) were also detected. The variations were attributed to dissimilar pathogen strains, environmental conditions and disease ratings conducted by different scorers at two sites. Inspection of individual TBBC3 lines in NY and NC respectively suggested that most of the lines performed similarly across the two locations. Pair-wise comparisons validated the phenotypic differences for seven out of the 15 TBBC3 lines and B73 (Table 2). Consistently in NY and NC, TBBC3-38 and TBBC3-39 were more resistant, whereas TBBC3-42, TBBC3-36, TBBC3-21, TBBC3-30, and TBBC3-77 were more susceptible than B73.

Ten selected TBBC3 lines showed significantly greater DTA than B73 (about 1-4 days of difference, Table 2), indicating the presence of flowering time QTL in those lines. To test the effect of plant development on QTL expression, the 15 selected lines were inoculated at juvenile and adult stages (Table 2) and their disease traits were compared. In all the lines, QTL effects in reducing or increasing resistance to NLB were generally consistent for juvenile and adult plant stages. No lines showed contrasting phenotypes (more resistant and more susceptible relative to B73) when inoculated at the five- to six-leaf stage or just before flowering, which suggested that allele effects of NLB QTL were not altered by developmental stage. There was, however, evidence suggesting that the effectiveness of QTL might change over plant development. The resistance in TBBC3-38, TBBC3-39, TBBC3-18 and TBBC3-75 was more effective in adult than in juvenile individuals. The formation of NLB lesions was delayed by around 0.5-2 days on mature plants of the same genotypes.

### Validation of QTL effects by linkage analysis

Each of the TBBC3 lines had more than one identified introgression from Tx303. To determine which of the introgressed regions in a given line was associated with resistance, several segregating BC_4_F_2 _populations were developed from crosses between selected TBBC3 lines (TBBC3-02, TBBC3-38, TBBC3-39, TBBC3-42, and TBBC3-77) and the recurrent parent B73. The BC_4_F_2 _individuals from each cross were evaluated for NLB resistance and genotyped using markers for the respective introgressed regions. Significant trait-marker associations were identified in all the BC_4_F_2 _populations under investigation (Additional file 3).

The QTL at bin 1.02 was found effective in two segregating populations: those derived from TBBC3-42 and TBBC3-77. Although the introgressed region extended from bin 1.01-1.02, higher significance was detected at the marker located at bin 1.02 so the QTL was designated *qNLB1.02*. In the BC_4_F_2 _derived from TBBC3-42, ~14% of the IP variation could be attributed to *qNLB1.02*. The B73 allele at *qNLB1.02 *was associated with slower disease development. Individuals homozygous for Tx303 alleles in bin 1.02 (replacement of *qNLB1.02_B73_*) were significantly more susceptible than B73. The Tx303 homozygotes showed lesions ~0.6 days earlier (lower IP; *P *< 0.0001) and lesions expanded more rapidly by ~0.35 mm/day (greater LE; *P *< 0.05) in the B73 × TBBC3-42 population. The same QTL acted differently in the B73 × TBBC3-77 population. While *qNLB1.02 *was effective in reducing DLA and AUDPC, no effect on IP was observed in the B73 × TBBC3-77 population. It is worth noting that TBBC3-42 is a much more susceptible genotype than TBBC3-77, which provided a better background for evaluating minor disease QTL.

The QTL at bin 1.06 was not identified based on previously reported genotype information. Although clear phenotypic variation was observed on IP and DLA in the BC_4_F_2 _populations from TBBC3-38 and TBBC3-39, no significant association was found between IP and the known introgressions at bins 1.03, 5.00, 5.01-5.03, and 5.07-5.09. The IP on BC_4_F_2 _individuals of TBBC3-38 and TBBC3-39 ranged from 11 to 18 dpi, while IP on B73 was generally between 12 and 15 dpi. Since these two populations displayed a greater variation in resistance, we inferred that a disease QTL was present in the population but not associated with a recognized introgression. To search for unrecognized introgressions, an additional 68 SSR markers across the genome were chosen to target chromosomal regions that were not well-covered by the original 130 RFLP and SSR markers on TBBC3 map. Pooled DNA samples from BC_4_F_2 _individuals of TBBC3-38 and TBBC3-39, along with B73 DNA as a control, were tested with the additional SSR markers for heterozygosity. Among the 68 SSR markers tested, *umc1754 *and *umc2234 *(both in bin 1.06) were the only polymorphic markers segregating in both BC_4_F_2 _populations. This indicated the presence of a Tx303 introgression at bin 1.06 in both TBBC3-38 and TBC3-39. Individuals in each population were then genotyped with *umc1754 *and *umc2234*, and analyzed for the effects on various disease components. A highly significant association was detected for various parameters in both populations (Additional file 3). In the B73 × TBBC3-38 and B73 × TBBC3-39 populations, around 33% and 53% (respectively) of the variation in AUDPC was explained by this QTL. Compared to the B73 allele, the Tx303 allele at *qNLB1.06 *increased the IP by ~2 days, decreased LE by ~0.3 mm per day, and reduced DLA by about 5-10%. Another QTL at bins 5.08-5.09 (Tx303 allele for resistance) was found in the B73 × TBBC3-38 population. The effect was relatively minor (*R^2 ^*≈ 0.05) compared to the *qNLB1.06_Tx303 _*identified in the same population.

QTL were also identified in bins 5.00, 5.01-5.02, and 8.03-8.05. Although contributing significant levels of resistance for IP or DLA in the B73 × TBBC3-77 and B73 × TBBC3-02 populations, the same introgressions/markers were not found effective in the populations derived from TBBC3-38, TBBC3-39 and TBBC3-42. The ambiguous results called the association into question, suggesting that the effectiveness of QTL at bins 5.00, 5.01-5.02, and 8.03-8.05 was affected by genetic background and/or environmental conditions.

### Validation of *qNLB1.02_B73 _*and *qNLB1.06_Tx303 _*in selected NIL sets

To confirm the QTL effects detected in segregating populations, NILs were generated by selfing selected BC_4_F_2 _lines from the populations B73 × TBBC3-38, B73 × TBBC3-39 and B73 × TBBC3-42. Selected lines were chosen to represent different introgressed regions in the original TBBC3 lines. The QTL effects were determined by pair-wise comparisons between individual introgression lines and B73, and between the NILs developed from a TBBC3 line. Within each set of NILs, lines contrasting for each marker locus were grouped and analyzed for their phenotypic differences. The NILs derived from different TBBC3 lines were not compared to each other since the phenotypic difference can be attributed to not only introgressed regions but also to minor differences in genetic backgrounds.

**Figure 2:**
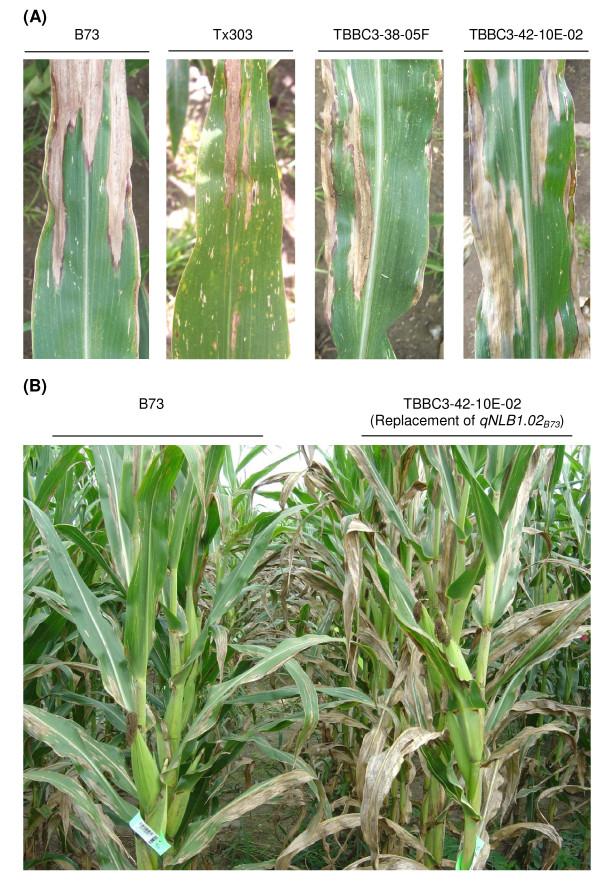
**Effects of *qNLB1.06_Tx303 _*and *qNLB1.02_B73 _*in the field**. (A) Different levels of NLB resistance in B73, Tx303, TBBC3-38-05F (the NIL carrying *qNLB1.06_Tx303_*), and TBBC3-42-10E-02 (the NIL carrying *qNLB1.02_Tx303_*, which is essentially "replacement of *qNLB1.02_B73_*"). The photographs were taken in Aurora NY, 2008, on the 7^th ^leaves at 28 days after inoculation. (B) Replacement of the B73 allele with the Tx303 allele at bin 1.02 largely increased the susceptibility to NLB. The photograph was taken in Aurora NY, 2008, at 68 days after inoculation. The comparison between B73 and TBBC3-38-05F is not shown because their difference in DLA (< 10%) is not differentiable in photographs.

Evaluations of the BC_4_F_3 _NILs validated the effects of *qNLB1.06_Tx303_*. Five NILs derived from B73 × TBBC3-38 and four NILs derived from B73 × TBBC3-39 were phenotyped in 2008. Individual trait-locus analysis in the two NIL sets revealed that bin 1.06 was the only locus that showed significant effects on resistance. The QTL effect at bin 5.08-5.09 was detected in the B73 × TBBC3-38 population, but was not confirmed in the NILs. As shown in Additional file 4, most of the NILs carrying Tx303 alleles at bin 1.06 were significantly more resistant than B73. The only exception was the line TBBC3-39-11A, which might have lost the resistance gene(s) due to recombination. Significant differences between lines with *qNLB1.06_Tx303 _*and B73 were observed for IP, DLA and AUDPC, which was in agreement with the results from linkage analysis in BC_4_F_2 _populations (Additional file 3). The effects of *qNLB1.06_Tx303 _*relative to *qNLB1.06_B73 _*on IP (1.5-2.5 days) and DLA (2-10% through the season) were determined by comparing TBBC3-38-05F with B73 (Figure 2A) and TBBC3-39-19E with B73. The significant effect of reducing lesion expansion was not confirmed in the NILs. Lines with *qNLB1.06_Tx303 _*even showed significantly higher LE scores, indicating some variations from different genetic backgrounds and environments.

*qNLB1.02 *was validated by analyzing six BC_4_F_3 _NILs and seven BC_4_F_4 _NILs derived from a B73 × TBBC3-42 cross. Evaluations conducted in 2007 and 2008 led to similar results. Since BC_4_F_4 _NILs had cleaner genetic backgrounds, the data from the 2008 trial was shown to represent the overall result. Individual trait-locus analysis in this NIL set suggested that bin 1.01-1.02 was the only locus affecting resistance. The NILs carrying Tx303 allele(s) at bin 1.01-1.02 were all significantly more susceptible than the ones carrying B73 allele(s) at the same region (Additional file 5), confirming the resistance effect of *qNLB1.02_B73_*. Highly significant differences between TBBC3-42-10E-02 (the NIL with *qNLB1.02_Tx303 _*in the cleaner background) and B73 were observed across all the disease components under investigation (Figure 2). The B73 allele(s) at *qNLB1.02 *delayed lesion formation by 1.6 days (*P *= 0.004), inhibited lesion expansion by 0.84 mm per day (*P *< 0.0001), and reduced DLA 18-38% through the season (*P *< 0.0001). Overall, the relative allele effects detected in the NILs were much greater than in the segregating populations, and the resistance of *qNLB1.02_B73 _*was more effective in the field than in the greenhouse.

### Characterization of *qNLB1.02_B73 _*and *qNLB1.06_Tx303 _*using derived NILs

Two derived NILs carrying identified NLB QTL in "cleaner" B73 backgrounds (minimal amount of introgression from Tx303 not associated with NLB resistance) were chosen for detailed QTL characterization. TBBC3-38-05F was a BC_4_F_3 _NIL with a Tx303 introgression at bin 1.06 (designated as the NIL with *qNLB1.06_Tx303_*), and TBBC3-42-10E-02 was a BC_4_F_4 _NIL with a Tx303 introgression at bin 1.02 (designated as the NIL with *qNLB1.02_Tx303_*). To investigate the effect of QTL during NLB development, a series of disease components (Table 1) was selected or developed and applied on the two derived NILs in three replicated experiments in the greenhouse during December 2007 - April 2008, and at Aurora, NY during summer 2008.

#### Microscopic investigation on the pathogenesis of *S. turcica*

**Figure 3:**
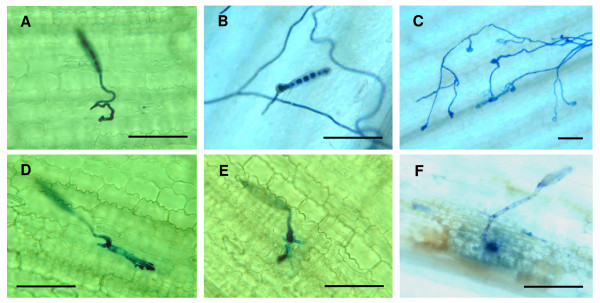
**Light micrographs of the infection and early colonization of *Setosphaeria turcica *in corn leaves**. Samples from greenhouse trials were stained with trypan blue. (A) A conidium germinated and formed an appressorium. (B) The penetration peg from the appressorium punched through the cuticle and epidermal cell wall. (C) The conidium could continue to produce new germ tubes and appressoria until exhausting its reserves or ultimately gaining entry into a plant cell. This phenomenon was occasionally observed on a resistant inbred line CML52. (D) A subcuticular palm-shaped structure occasionally developed from the penetration peg (likely before hyphae infected the epidermal cell). (E and F) Cytoplasmic depletion of the conidium was seen after the infection process. Infective hyphae spread from primary infected cell to surrounding area, causing host cell death. (Infected leaf samples of A, D and E: CML52, 2dpi; B: B73, 2 dpi; C: CML52, 3 dpi; F: IBM262, 5 dpi) (Scale bars, 100 μm)

**Figure 4:**
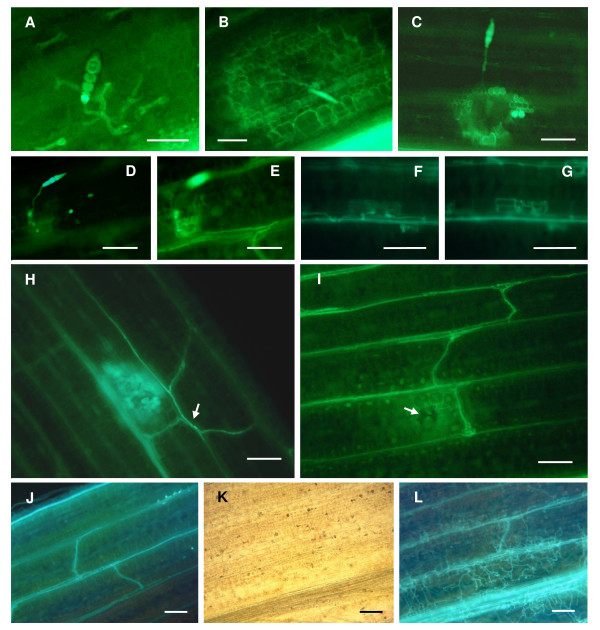
**Fluorescence micrographs of the pathogenesis of *Setosphaeria turcica *in corn leaves**. Samples from greenhouse trials were treated with KOH-aniline blue. (A) A germinated conidium on leaf surface. (B) Infective hyphae grew into contact with mesophyll cells. (C and H) Defense responses induced around the infection site. The brightly fluorescing area was presumably caused by callose deposition and the accumulation of autofluorescent phenolic compounds. The fluorescing vascular bundles (arrow), possibly due to lignification, could be differentiated from the hyphae growing in the vasculature by the lack of distinguished hyphal coils. (D and E) Infective hyphae grew towards the vascular bundle and invaded it. D and E represent different focal planes. (F and G) Hyphae grew out the vascular vessel to colonize the neighboring bundle sheath cells. F and G represent different focal planes. (I) Hyphae successfully spread through the vascular system. The weak fluorescence and collapsed cells surrounding the infection site (arrow) were typical symptoms of the compatible interaction. (J) Movement of infective hyphae through vascular bundles and cross veins. Leaf tissue remained non-wilted at this stage. (K and L) Colonization of necrotic hyphae in the wilted lesion. Vascular bundles were plugged with aggressively growing hyphae. Hyphae branched out from the colonized vasculature to the rest of the leaf. K and L were viewed in the light- and fluorescent-field, respectively. (Infected leaf samples of A: Tx303, 4 dpi; B: CML52, 4 dpi; C: B73, 4 dpi; D and E: TBBC3-42, 4 dpi; F and G: CML103, 7 dpi; H: B73, 4 dpi; I to L: B73, 10 dpi) (Scale bars, 100 μm)

Trypan blue staining and KOH-aniline blue fluorescence microscopy techniques were used for histological examination of NLB development *in planta*. To understand typical resistant and susceptible interaction patterns in the NLB pathosystem, preliminary microscopic analysis was conducted on the following maize lines challenged with *S. turcica*: B73, Tx303, TBBC3-38, TBBC3-42, the resistant maize inbred line CML52 and CML103, and a susceptible recombinant inbred line IBM262. As illustrated in Figures 3 and 4, pathogenesis was successfully visualized and can be summarized as follows. After landing and attaching on the leaf surface of a susceptible maize plant, the conidium of *S. turcica *germinates. It forms an appressorium, which produces a penetration peg that punches through the cell wall and into the epidermal cell. From this cell, infective hyphae are produced, grow towards the vascular bundle, enter the vasculature and ramify within it. Aggressive hyphal growth and extension in the vascular bundles can be seen. The hyphae can grow for several days inside the vascular bundles without causing visible symptoms (incubation period was approximately seven days on susceptible maize lines). After extensive growth within the vascular veins, hyphae grow out to colonize the neighboring bundle sheath cells and then branch out to colonize the rest of leaves. This progression is consistent with several previous studies on *S. turcica *pathogenesis on maize [23-25]. The limited damage *S. turcica *causes to host tissues at early phase of pathogenesis and the extended incubation period suggests the possibility that this is a hemibiotrophic interaction.

Among diverse maize genotypes (B73, Tx303, CML52, CML103 and IBM262) and on genotypes carrying different QTL for resistance (B73, TBBC3-38, and TBBC3-42), differential phenotypes were observed for the timing and extent of certain steps in the processes of infection and colonization. To document these differences, a series of microscopic parameters was chosen for characterizing NLB QTL efficacy at different stages during pathogenesis. The microscopic disease components used in the study included the number of appressoria, infection efficiency, vascular invasion efficiency, the size of strongly fluorescent area surrounding the infection site, and the appearance of necrotic or fortified vascular bundles. The microscopic evaluations on B73, Tx303 and the derived NILs were performed on plants grown in controlled greenhouse conditions as well as in the field.

#### QTL effect on the infection of *S. turcica*

**Figure 5:**
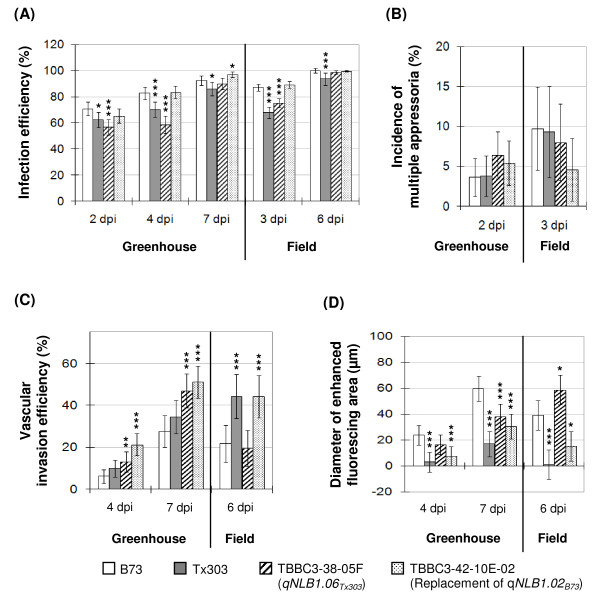
**Investigation of QTL effects on microscopic disease components**. Microscopic disease components including (A) Infection efficiency, (B) incidence of multiple appressoria, (C) vascular invasion efficiency, and (D) size of strongly fluorescing area surrounding the infection site, were used to assess QTL effects in controlled greenhouse condition and field. Infected leaf samples were collected from maize genotypes B73 (open), Tx303 (gray), TBBC3-38-05F (hatched; the NIL carrying *qNLB1.06_Tx303_*) and TBBC3-42-10E-02 (dotted; the NIL carrying *qNLB1.02_Tx303_*, which is essentially "replacement of *qNLB1.02_B73_*"). Samples collected 2 days post inoculation (dpi) from greenhouse and 3 dpi from field were stained with trypan blue, while the samples collected 4 dpi and 7 dpi from greenhouse and 6 dpi from field were treated with KOH-aniline blue fluorescence technique. Differences between least square means of different genotypes relative to B73 were determined by two-tailed Student's t test (significance level: * 0.01 <*P *< 0.05; ** 0.001 <*P *< 0.01; *** *P *< 0.001). In graphs A - C, the proportion data were arcsine transformed for statistical analysis, and the corresponding least squares means and 95% confidence intervals were back transformed to original scale before plotting. The confidence intervals are bigger than significance levels due to asymmetry resulting from back transformation.

Infection efficiency was examined by trypan blue and KOH-aniline blue staining, followed by scoring the number of conidia that had successfully penetrated the epidermal cell walls. The infection efficiency was defined as the ratio of successful infection sites over the total number of germinated conidia. Non-germinated conidia were excluded because of the difficulty of determining whether a non-germinated conidium was viable, given the fact that a small proportion of damaged conidia was always present in the spore suspension. In both the greenhouse and field, lower infection efficiency was consistently observed on the NIL with *qNLB1.06_Tx303 _*at earlier time points examined, as well as on Tx303 at all time points examined (Figure 5A), compared with B73. In the greenhouse, *qNLB1.06_Tx303 _*reduced infection efficiency by ~14% at 2 dpi and by ~24% at 4 dpi (*P *< 0.0001). Similarly, in the field, infection efficiency was reduced by ~12% at 3 dpi (*P *< 0.0001) relative to B73. The resistant parental genotype Tx303 showed a stronger effect on reducing the infection of *S. turcica *in the field (decreased by ~19% relative to B73, *P *< 0.0001) than in the greenhouse (decreased by 6.5-12.5% relative to B73, *P *< 0.05). No significant effect on pathogen penetration was seen from *qNLB1.02_Tx303_*.

The incidence of multiple appressoria per germinated conidium was used as another indicator for resistance to fungal penetration. It was observed that when a germinated conidium failed to penetrate the epidermis, the conidium would continue to produce new germ tubes and appressoria until exhausting its reserves or ultimately gaining entry into a plant cell (Figure 5B). Despite the effect of *qNLB1.06_Tx303 _*on reducing the penetration of *S. turcica*, no difference was detected on the formation of multiple appressoria among B73, Tx303, and the derived NILs.

#### QTL effect on the vascular invasion of *S. turcica*

Intracellular hyphal growth from the initially infected epidermal cell to surrounding mesophyll cells, and the subsequent invasion of the vascular bundle, were investigated by KOH-aniline blue fluorescence microscopy. Vascular invasion efficiency was defined as the incidence of hyphal growth into the vasculature per infection site. Differences in vascular invasion efficiency were observed between B73 and the other genotypes (Figure 5C). The NIL with *qNLB1.02_Tx303 _*had greater vascular invasion efficiency, indicating the superiority of the B73 allele(s) at this locus. Replacement of the B73 allele with the Tx303 allele at *qNLB1.02 *(in lines carrying an introgression from Tx303 at this locus) led to 15-24% of increase in the incidence of vascular invasion in both greenhouse and field (*P *< 0.0001). The *qNLB1.06_Tx303 _*allele(s) was not effective for inhibiting hyphal growth into the vasculature. Under greenhouse conditions, the NIL with *qNLB1.06_Tx303 _*was observed to have a 7-19% higher susceptibility for vascular invasion (*P *< 0.0001), which is a further indication that this QTL functions at the stage of penetration but not at later stages of pathogenesis. The susceptibility to vascular invasion was also seen in Tx303. In the greenhouse, higher vascular invasion efficiencies were observed in Tx303 than in B73, although with marginal significance levels. In the field, the invasion efficiency in Tx303 was ~22% greater than in B73 (*P *< 0.0001).

#### QTL effects on the accumulation of defense compounds around the infection site

Infected tissues stained with aniline blue were also used for revealing defense responses induced upon pathogen challenge. The fluorescence emitted from the cells surrounding the infection site is presumably due to callose deposition [60,61] and the accumulation of autofluorescent phenolic compounds [62-64]. Callose and phenolics are antimicrobial compounds that help restrict the establishment and spread of *S. turcica *in the leaf. In susceptible genotypes, weak fluorescence and collapsed cells were generally observed around the infected sites (Figure 4I), suggesting the lack of induced defense responses. The more resistant genotypes showed enhanced fluorescence from localized cell regions (Figure 4C and 4H).

To assess the degree of defense response around the primary infected cell, the diameter of brightly fluorescing area was measured under a fluorescence microscope with a micrometer (Figure 5D). The average diameter of the fluorescing area in the NIL with *qNLB1.02_Tx303 _*was consistently smaller than that seen for B73 (*P *< 0.0001 in greenhouse, *P *= 0.02 in the field), indicating that the B73 allele(s) at *qNLB1.02 *contribute to the regulation, production or accumulation of callose, lignin or other phenolic compounds. Interestingly, compared to B73, the fluorescing area in the NIL with *qNLB1.06_Tx303 _*was slightly larger (*P *= 0.052) in the field, but was significantly smaller (*P *< 0.0001) at 7 dpi in the greenhouse, suggesting that the phenotype conferred by *qNLB1.06_Tx303 _*is vulnerable to environmental influences.

Considering the potential role of vascular resistance in the NLB pathosystem, the incidence of brightly fluorescing vascular bundles (likely caused by lignification) was also evaluated. Although this response was pronounced in CML52 (Figure 4H), this type of resistance was rarely observed in B73, Tx303 and the derived NILs. The induced defense reaction around the infection site was fairly weak at all times examined in either greenhouse or field conditions. It was thus not considered a relevant parameter for testing the effect of *qNLB1.06_Tx303 _*and *qNLB1.02_B73_*.

#### QTL effect on mycelial growth of *S. turcica *in planta

**Figure 6:**
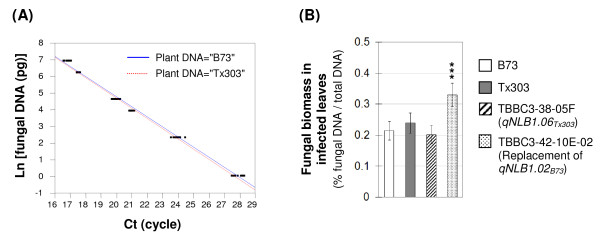
**Quantifying QTL effect on mycelial growth of *Setosphaeria turcica in planta *using DNA-based real-time PCR**. (A) Two standard curves were constructed by mixing a series of *S. turcica *DNA and 50 ng of maize DNA from non-inoculated B73 and Tx303 plants, respectively. With B73 DNA, Ln[fungal DNA] = 16.912 - 0.606*Ct (*R^2 ^*= 0.99); with Tx303 DNA, Ln[fungal DNA] = 16.906 - 0.610*Ct (*R^2 ^*= 0.99). Ln: natural logarithm. Ct: threshold cycle, the number of PCR amplification cycle at which the exponential increase of the product was detected. (B) Measurement of *S. turcica *DNA ratio in infected leaves collected 9 dpi from maize genotypes B73 (open), Tx303 (gray), TBBC3-38-05F (hatched; the NIL carrying *qNLB1.06_Tx303_*) and TBBC3-42-10E-02 (dotted; the NIL carrying *qNLB1.02_Tx303_*, which is essentially "replacement of *qNLB1.02_B73_*"). Differences between least square means of different genotypes relative to B73 were determined by two-tailed Student's t test (significance level: * 0.01 <*P *< 0.05; ** 0.001 <*P *< 0.01; *** *P *< 0.001). The proportion data were arcsine transformed for statistical analysis, and the corresponding least squares means and 95% confidence intervals were back-transformed to original scale before plotting. The confidence intervals are bigger than significance levels due to asymmetry resulting from back transformation.

DNA-based qPCR was developed to precisely quantify mycelial growth of *S. turcica *in maize leaves. The *R*-square values for the two standard curves based on B73 and Tx303 respectively were both higher than 0.99 (Figure 6A), indicating that the designed ITS primer pair had good sensitivity and specificity for reliably amplifying fungal DNA. To assess the qPCR technique as a tool for analyzing the NLB pathosystem, several time-course experiments were performed on seven maize genotypes with a wide range of differential levels of NLB resistance (C. Chung, unpublished). Preliminary results showed that the levels of fungal DNA ratios determined by qPCR approximately conformed to the performance of resistance in the field. For maize genotypes with low to intermediate levels of resistance, fungal DNA was detected as early as three days after inoculation. The detectable fungal DNA ratios increased over time until the end of the incubation period (when visible lesions formed), and thereafter decreased. This reduction in pathogen DNA was unexpected, and apparently corresponded to a loss of DNA integrity. DNA samples extracted from tissues showing lesions were usually brownish and of poor quality. The decrease in detectable fungal DNA ratio in highly diseased leaves is contradictory to our microscopic observation of abundant mycelial growth in NLB lesions. It is likely that in tissues with actively developing lesions, more DNA-degrading enzymes involving in cell death are present and can digest fungal DNA during the extraction. To better differentiate resistant and susceptible responses, it is critical to apply qPCR on infected tissues taken prior to the appearance of necrotic lesions on the most susceptible genotype in an experimental set.

Infected leaves from B73, Tx303, and two derived NILs were collected nine days after inoculation (according to the IP of the susceptible NIL), and measured for their fungal DNA content using qPCR. As shown in Figure 6B, neither Tx303 nor *qNLB1.06_Tx303 _*showed significant effect on reducing the growth of *S. turcica *in leaves. However, the fungal biomass ratio (% of fungal DNA in the infected leaf) in the NIL with *qNLB1.02_Tx303 _*was 24% higher than in B73 (*P *< 0.0001), indicating that the *in planta *development of *S. turcica *is more extensive on plants without *qNLB1.02_B73_*.

### Preliminary characterization of potential QTL effects for multiple disease resistance using derived NILs

Significant differences were observed between Tx303 and B73 for resistance to Stewart's wilt, anthracnose stalk rot (ASR), common smut and common rust. In the field, Tx303 was more resistant to Stewart's wilt and ASR, while B73 was more resistant to common smut and common rust. To investigate the resistance spectrum of *qNLB1.02 *and *qNLB1.06*, several NILs derived from B73 × TBBC3-38, B73 × TBBC3-39 and B3 × TBBC3-42 were evaluated for the four diseases (Additional files 4 and 5). The choice of NILs was constrained by the availability of seeds.

The evidence suggested that both *qNLB1.06_Tx303 _*and *qNLB1.02_B73 _*were effective for resistance to Stewart's wilt. As shown in Additional file 4, all the NILs carrying Tx303 introgression at bin 1.06 were significantly more resistant than B73. Based on comparing TBBC3-39-19E to B73, *qNLB1.06_Tx303 _*was inferred to be effective for reducing lesions of Stewart's wilt on inoculated leaves by as much as 39% (*P *< 0.0001). Likewise, in Additional file 5, lines with Tx303 alleles at bin 1.01-1.02 were significantly more susceptible than B73. The effect of *qNLB1.02_B73 _*on Stewart's wilt was estimated as reducing 19% of primary DLA based on a comparison of TBBC3-42-10E-02 and B73 (*P *< 0.0001). Significant differences were also observed among the lines fixed (homozygous for Tx303 or B73 alleles) at bins 1.01-1.02, indicating that there are QTL other than *qNLB1.02_B73 _*segregating in the NIL set. However, none of the rest of the introgressed regions were unambiguously associated with resistance.

*qNLB1.06_Tx303 _*was ineffective for resistance to ASR, smut and rust. No phenotypic difference in the development of rust and smut galls was detected among B73, TBBC3-38, TBBC3-39 and their derived NILs. Significant difference in discolored internode area was observed between TBBC3-38 and B73 (~32%, *P *= 0.001), but the variation was not associated with *qNLB1.06*, as TBBC3-39 and the NIL carrying a single introgression at bin 1.06 (TBBC3-39-19E) showed the same resistance level as B73 (Additional file 4).

*qNLB1.02_B73 _*was ineffective for resistance to ASR and common smut, but potentially effective for resistance to common rust (Additional file 5). For ASR, some phenotypic difference was found among B73, TBBC3-42 and derived NILs, but none of the identified introgressed regions were associated with resistance. B73 was more resistant than Tx303 for common smut and common rust. For common smut, there were almost no ear galls or stalk galls observed on B73, TBBC3-42 and TBBC3-42-10E-04, suggesting that introgressed segments in TBBC3-42 did not affect smut development. (More ear galls were scored on TBBC3-42-10E-04 in the 2008 trial, but the significance was marginal (*P *= 0.04), and the finding was not consistent with the 2007 result). For common rust, except for TBBC3-42-06F-2, lines with Tx303 alleles at bin 1.01-1.02 were consistently more susceptible than B73 for all three severity ratings (differed by 1.1-3.1 scales in severity, *P *< 0.025) and AUDPC (differed by about 43-53 scale-day, *P *< 0.001). The result indicated the possibility of rust QTL at bins 1.01-1.02 and/or unidentified introgressed regions in TBBC3-42.

## Discussion

### Identification of NLB QTL using TBBC3 introgression lines

Two QTL for resistance to NLB, *qNLB1.02_B73 _*and *qNLB1.06_Tx303_*, were successfully identified, validated and characterized using a population of introgression lines and derived NILs. We applied a stepwise strategy that allowed phenotyping of informative NILs over a series of generations. A special field design, in which each row of CSSL/NIL arranged next to a row of B73, allowed accurate visual comparisons and a relatively uniform epidemic across the field. We detected and sequentially validated QTL using multiple disease components, in a full set of 82 lines, a subset of 15 selected lines, five selected BC_4_F_2 _populations, and three sets of derived BC_4_F_3_/BC_4_F_4 _NILs. Our primary hypothesis that individual QTL affect distinct stage(s) of NLB development was ultimately confirmed using two selected NILs (compared to B73) in repeated greenhouse and field trials, with a panel of conventional and novel components. Our results revealed that *qNLB1.06_Tx303 _*and *qNLB1.02_B73 _*are mainly effective against the infection and colonization of *S. turcica*, respectively. We found that the QTL were both effective in both juvenile and adult plants, and that both chromosomal segments were associated with resistance to more than one disease.

The mechanism underlying resistance conferred by *qNLB1.02_B73 _*and *qNLB1.06_Tx303 _*is unlikely to be the same as the mechanism underlying qualitative resistance conferred by currently known major genes. Qualitative resistance to NLB is generally characterized by chlorotic-necrotic lesions [65-69], chlorotic halo lesions [70], or extremely prolonged IP [71]. Considerable levels of resistance were seen for *qNLB1*.*02_B73 _*and *qNLB1.06_Tx303 _*in the two field sites, without observing distinct lesion type or much greater IP. Moreover, although co-localized NLB QTL at bins 1.02 and 1.06 have been reported in other populations [72,73], no major genes have been mapped to these chromosomal regions.

### Conventional and newly-developed components of resistance targeting different stages of NLB development

QTL effects were analyzed using five conventional macroscopic disease parameters and four microscopic parameters. Each of the macroscopic components reflected different phase(s) of disease development. Incubation period quantifies the time to appearance of wilted lesions, which reflects the speed of xylem plugging due to extensive hyphal growth in the veins. Lesion expansion quantifies the rate of expansion of wilted and necrotic lesions, which reflects the speed of destructive hyphal growth in the leaves. Ratings for diseased leaf area, disease severity and area under the disease progress curve, on the other hand, involve visual quantification of overall disease progress on the entire plant. In our greenhouse and field trials, IP showed a better correlation than LE with DLA, disease severity and AUDPC. This indicates that IP is a more discriminating parameter than LE for measuring NLB severity. This confirms previous reports that IP is a convenient target trait in selection and breeding for resistance to NLB [11,73-78]. In a recurrent selection study for NLB resistance, selection for prolonged IP resulted in ~7.5% more gain in reducing AUDPC per selection cycle compared to selection for lesion length [11].

QTL effects were also microscopically investigated at the pre-penetration, infection, and colonization phases. In most fungal pathosystems, pre-penetration resistance is associated with specialized physical and chemical features of plant surface which help reduce the incidence of landing, adhesion, germination, appressorium formation and penetration of pathogenic fungi [79]. Post-penetration resistance to microbial attack, on the other hand, is characterized by programmed necrosis, along with the induction of callose, lignin, phenolic compounds and other pathogenesis-related proteins around the primary-infected cell. Four microscopic components, including the incidence of multiple appressoria, infection efficiency, accumulation of defense materials surrounding the infection site, and vascular invasion efficiency, were developed to complement the conventional macroscopic components. Modified trypan blue staining [25,42] and KOH-aniline blue fluorescence [43] methods were useful in determining the degree and timing of allelic contribution to quantitative resistance to NLB. Previous studies utilizing sectioning and electron microscopy have revealed that xylem plugging is a key stage for the formation of NLB lesions (Jennings and Ullstrup, 1957). Our microscopic examination using KOH-aniline blue fluorescence technique confirmed the finding and allowed the observation of *in planta *colonization of *S. turcica *from a different angle. In all the maize genotypes examined, the mycelium appeared to grow preferentially towards vascular bundle, initially invading the vasculature, then extending through the xylem vessel, and eventually, aggressively growing out to the neighboring mesophyll tissues. To our knowledge, the mechanisms of the post-infection directional growth of vascular fungal pathogens remain to be elucidated.

qPCR was employed for the first time for the measurement of *in planta *biomass of *S. turcica*. Although it has been widely applied in other pathosystems, qPCR can only be used for NLB quantification at early stages of pathogenesis. The accuracy and differentiating power are highly dependent on uniform inoculation and precise sampling at the early stages of pathogenesis, so the method is best suited to work conducted under controlled conditions. The biggest constraint of this technique comes from its not being able to measure the destructive mycelial growth in the blighted leaf areas, possibly due to the poor quality of DNA extracted from necrotic tissues. The cost of qPCR reagents and the required workload make it unfeasible for regular phenotypic screening. However, qPCR serves as a good tool for quantifying the degree of earlier mycelial colonization of *S. turcica *in maize leaves.

### Hypothesis #1: individual QTL affect distinct stages of disease development

Detailed macroscopic and microscopic evaluations revealed the distinct features of *qNLB1.06_Tx303 _*and *qNLB1.02_B73 _*in NLB development. *qNLB1.06_Tx303 _*conditions resistance mainly against the penetration of *S. turcica*. The anti-penetration effect was observed at earlier but not later time points examined, indicating that *qNLB1.06_Tx303 _*acts in a quantitative manner, which delays rather than prevents the occurrence of infection. In contrast, *qNLB1.02_B73 _*appears to condition resistance not to infection, but rather by enhancing the induction of defense reactions surrounding infection sites, as well as by inhibiting hyphal growth into the vascular bundle, and the subsequent necrotrophic colonization in the leaves. It is difficult to infer whether the resistance is associated with defense reactions in mesophyll, parenchyma, bundle sheath and/or other cells.

Delaying the invasion and extension of *S. turcica *in the vascular system was shown to be critical for quantitative resistance to NLB. This is consistent with previous microscopic analyses [23,24] suggested that xylem invasion/plugging is an important pathogenetic phase in NLB progress. Polygenic resistance (in an inbred line CI90A) has been associated with reduced hyphal spread into the xylem [22]. The significance of protecting the vasculature from hyphal invasion is supported by the evidence that the replacement of superior B73 allele with Tx303 allele at *qNLB1.02 *led to an 18-38% higher DLA (relative to B73). Incorporating a QTL effective in slowing down the invasion of the vasculature would thus considerably increase overall resistance.

We speculate that preventing the attachment of conidia and the subsequent infection plays a role in quantitative resistance to NLB in the donor line Tx303. Although this was not quantified, it was visually obvious that the number of spores per leaf segment of Tx303 was lower than the spore numbers on B73 or the NILs carrying *qNLB1.06_Tx303 _*and *qNLB1.02_B73_*. Lower infection efficiency of *S. turcica *was observed for Tx303 relative to B73. Interestingly, Tx303 showed a greater susceptibility than B73 for the parameters of vascular invasion efficiency, enhanced fluoresced area, and lesion expansion. Despite the lack of resistance in delaying vascular invasion and extension of *S. turcica *in Tx303, its higher effectiveness in reducing spore attachment and infection may contribute to the overall moderate resistance to NLB.

### Hypothesis #2: the effectiveness of disease QTL is affected by plant maturity

A number of maize diseases caused by hemibiotrophic fungi (eg. gray leaf spot, anthracnose leaf blight, anthracnose stalk rot) and necrotrophic fungi (eg. southern leaf blight) are known to progress more rapidly on the plants after anthesis [18,49,80,81]. In maize, a significant correlation has been detected between disease resistance and flowering time in a panel of 253 diverse lines (R. Wisser, J. Kolkman, P. Balint-Kurti, and R. Nelson, unpublished). The fact that flowering time may account for a considerable proportion of resistance variation leads to the hypotheses that the effects of disease QTL reflect indirect expression of flowering time QTL, and/or that the effects of disease QTL are modulated differently at varied plant developmental stages. In fact, several QTL identified in biparental populations are associated with both NLB resistance and flowering time (J. Poland, pers. comm.).

The association between NLB QTL and plant maturity was investigated in the subset of 15 selected TBBC3 lines. To separate resistance effects from maturity effects, plants in two different fields were inoculated at juvenile and adult stage (two weeks before tasselling) respectively, and evaluated for NLB resistance and flowering time. Generally consistent expression of resistance or susceptibility was observed at the two stages, implying that the effectiveness of these QTL was not altered substantially by plant maturity. While several of the introgression lines (10 out of the 15 TBBC3 lines with differential resistance relative to B73) showed significantly different flowering times relative to B73, the interactions between NLB QTL and flowering time QTL were relatively minor. For the *qNLB1.02_B73 _*and *qNLB1.06_Tx303_*, the analysis of advanced NIL sets further provided strong evidence of their independence from flowering time. This agrees with a previous report of flowering-time QTL in TBBC3 introgression lines [27]: bins 1.01-1.02 and 1.06 were not among the QTL affecting days to anthesis, days to silking, or anthesis-silking interval.

### Hypothesis #3: the effectiveness of QTL is affected by environmental conditions

A major limitation for QTL applications is the lack of consistency of QTL effects across environments. The inconsistent detection of QTL has been associated with experimental errors and differential gene expression affected by environmental factors [82]. In view of the widely reported inconsistency, the present study revealed that the introgressions/QTL with relatively large effects (carried in seven introgression lines: TBBC3-38, TBBC3-39, TBBC3-42, TBBC3-36, TBBC3-21, TBBC3-30, and TBBC3-77) were consistent in their performance across field sites and years. Reliable expression of these QTL, including *qNLB1.02_B73 _*and *qNLB1.06_Tx303_*, suggests their applicability to resistance breeding.

Disease resistance can be expressed differentially, not only in different field environments but also under field versus greenhouse conditions (eg. [80,83,84]). Differential efficacy of *qNLB1.02_B73 _*and *qNLB1.06_Tx303 _*in controlled greenhouse and field conditions was evaluated using macroscopic and microscopic phenotypes. It should be noted that due to the different inoculation and sampling treatments necessarily employed in different environments, the comparison between the effectiveness of disease QTL in the greenhouse and field should be addressed based on relative observation rather than the absolute values. Our overall results suggest that the resistance of *qNLB1.02_B73 _*was stably expressed in the greenhouse and field, whereas *qNLB1.06_Tx303 _*conferred a higher level of resistance in the field than in the greenhouse. Macroscopically, the effects of *qNLB1.06_Tx303 _*on IP and primary DLA in greenhouse-grown plants were occasionally insignificant. Microscopically, the positive effect of *qNLB1.06_Tx303 _*on triggering defense response surrounding the initially infected cell was only detected in the field. Greenhouse-grown plants carrying *qNLB1.06_Tx303_*, however, displayed an opposite effect (smaller fluorescent area), which suggests that this type of resistance is highly affected by environmental factors. The observation that *qNLB1.06_Tx303 _*increased the efficiency of *S. turcica *invasion into vasculature in the greenhouse but not field, may be an indirect effect of decreased accumulation of host defensive materials at the post-infection stage.

### Hypothesis #4: QTL for resistance to NLB comprises genes or gene clusters involving in broad-spectrum resistance

The phenomenon of multiple disease resistance (MDR) has been inferred based on the detection of QTL clusters affecting different diseases [2,19,20,85], genetic correlations in populations [86], non-specific defense mechanisms (eg. SAR [87-89]), and genes (eg. *npr1 *[90] and *mlo *[91]). Maize bins 1.02 and 1.06 have been previously associated with a number of disease QTL mapped in diverse maize populations [20]. Our evaluations of resistance to common rust, Stewart's wilt, anthracnose stalk rot, common smut, and common rust in sets of NILs suggest that both *qNLB1*.*02_B73 _*and *qNLB1.06_Tx303 _*encompass gene(s) contributing nonspecific defense effects. *qNLB1*.*02_B73 _*was effective in decreasing NLB, common rust and Stewart's wilt, while *qNLB1.06_Tx303 _*was effective in decreasing NLB and Stewart's wilt. Nevertheless, due to the low resolution of QTL localization in this study, it is unclear whether the nonspecific resistance of *qNLB1*.*02_B73 _*and *qNLB1.06_Tx303 _*is caused by pleiotropy or linkage, or whether their effects are mongenic or polygenic. Although the underlying mechanisms are unknown, the feature of broad-spectrum resistance makes the two identified disease QTL appealing in practical applications.

It is worth noting that the NILs carrying *qNLB1.06_Tx303 _*showed remarkable resistance against Stewart's wilt, suggesting the involvement of major gene effect. In fact, a dominant major gene locus for Stewart's wilt, *Sw1*, has been mapped to bin 1.05-1.06 in inbred line Ki14 [92,93]. The co-localization of QTL for resistance to NLB and Stewart's wilt has also been observed in the NILs derived from the inbred line CML52 crossed to B73 (CML52 allele for resistance; C. Chung, unpublished).

## Conclusions

Our research has led to successful identification of two reliably-expressed QTL that can potentially be utilized to protect maize from *S. turcica *in different environments. Map-based cloning will reveal more about the genes and mechanisms underlying the distinct features of *qNLB1*.*02_B73 _*and *qNLB1.06_Tx303 _*in the pre-penetration, penetration and post-penetration phases of pathogenesis. Large mapping populations have been generated from the NILs and fine-mapping of *qNLB1*.*02_B73 _*and *qNLB1.06_Tx303 _*is in progress.

## Authors' contributions

CC and RN conceived of the study. CC designed and carried out all the experiments in New York, conducted statistical analysis, and drafted the manuscript. JL and EW contributed to the design of microscopic analysis, participated in phenotypic data collection in the greenhouse and field trials in NY during 2007-2008. ZK participated in the inoculation and phenotypic data collection in the field trials in NY in 2008. GVE carried out the field trials in NC. PBK designed and carried out the field trials in NC, participated in coordination of the study, and contributed to its revision. RN participated in the coordination of the work and the experimental design, helped to draft the manuscript and contributed to its revision. All authors read and approved the final manuscript.

## Supplementary Material

Additional file 1 **NLB resistance of the full set of 82 TBBC3 introgression lines**. Relative area under the disease progress curve (Relative AUDPC) values shown are the differences of least squares means (from mixed models) between TBBC3 lines and B73 recurrent parent. AUDPC was calculated from three diseased leaf area (DLA) scores in the 2006 trial in NY (solid bars), or three disease severity scores in the 2006 trial in NC (open bars). In NY, primary DLA was also rated for diseased leaf area on inoculated leaves. The letters "R" and "S" below the graph indicate the lines significantly more resistant and more susceptible than B73 at *P *< 0.05, respectively, based on primary DLA and AUDPC. The 15 TBBC3 lines selected for subsequent phenotypic validation are indicated by rectangles highlighting the maize line designation. Click here for file

Additional file 2 **Putative NLB QTL identified in the TBBC3 population**. Putative QTL for northern leaf blight (NLB QTL) affecting incubation period (IP), primary diseased leaf area (PrimDLA), diseased leaf area (DLA), disease severity (severity), and AUDPC (area under the disease progress curve calculated from DLA or disease severity) were identified using 82 TBBC3 introgression lines. QTL effects for each marker locus are the significant differences of least squares means of Tx303 homozygous genotypes at the locus relative to B73 recurrent parent line (* 0.01 <*P *< 0.05, ** 0.001 <*P *< 0.01, *** *P *< 0.001). Putative QTL are reported as correlated groups because of the high dependencies among those introgressed segments in TBBC3 lines. Click here for file

Additional file 3 **Validation of NLB QTL in the BC_4_F_2 _segregating populations**. The BC_4_F_2 _populations were genotyped and phenotyped for incubation period (IP), lesion expansion (LE), diseased leaf area (DLA), and area under the disease progress curve (AUDPC). The trait-marker association was tested by ANOVA at *P *< 0.05. Introgressions/markers significantly associated with NLB resistance are listed. The relative allele effects are the differences on the least squares means (LSMean) between Tx303 homozygous genotypes and B73 homozygous genotypes at the locus. Click here for file

Additional file 4 **Genotypes and disease phenotypes for Tx303, B73 and the NIL sets derived from B73 × TBBC3-38 and B73 × TBBC3-39**. Among the target introgressions at bins 1.03, 1.06, 3.02, 5.00, 5.02-5.03 and 5.07-5.09, only *qNLB1.06_Tx303 _*(Tx303 allele at bin 1.06) was validated for association with resistance to NLB. The open bars and solid bars represent the loci homozygous for B73 alleles and Tx303 alleles, respectively. The gray bars represent heterozygous loci or missing genotypic data. Only the chromosomes with introgressed regions in the two NIL sets are shown. The rest of the genome was assumed fixed for B73 alleles. Trait values are least squares means calculated from the mixed model. Pair-wise Student's t tests were performed to analyze the differences between each NIL and B73, and between every pair of NILs in each set. Trait values with different letters are significantly different from each other. Disease phenotypes that were significantly more resistant than B73 are highlighted in bold and shaded, while the phenotypes significant more susceptible than B73 are underscored. Lines that showed significantly different days to anthesis are in bold italic. *qNLB1.06_Tx303 _*was also effective for resistance to Stewart's wilt. (IP: incubation period; LE: lesion expansion; DLA: diseased leaf area; PrimDLA: primary DLA; AUDPC: area under the disease progress curve) Click here for file

Additional file 5 **Genotypes and disease phenotypes for Tx303, B73 and the NIL set derived from B73 × TBBC3-42**. Among all target introgressions at bins 1.01-1.02, 4.06-4.07, 5.02-5.03, 7.01, 8.02 and 8.03-8.05, only *qNLB1.02_B73 _*(B73 allele at bin 1.02) was validated for association with resistance to NLB. The open bars and solid bars represent the loci homozygous for B73 alleles and Tx303 alleles, respectively. The gray bars represent heterozygous loci or missing genotypic data. Only the chromosomes with introgressed regions in the NIL sets are shown. The rest of the genome was assumed fixed for B73 alleles. Trait values are least squares means calculated from the mixed model. Pair-wise Student's t tests were performed to analyze the differences between each NIL and B73, and between every pair of NILs. Trait values with different letters are significantly different from each other. Disease phenotypes that were significantly more resistant than B73 are highlighted in bold and shaded, while the phenotypes significant more susceptible than B73 are underscored. Lines that showed significantly different days to anthesis are in bold italic. Preliminary evidence also suggested that *qNLB1.02_B73 _*was effective for resistance to Stewart's wilt, and QTL at bin 1.01-1.02 and/or 5.02-5.03 were associated with resistance to common rust. Click here for file
