# Supplementary material for: Resistance loci affecting distinct stages of fungal pathogenesis: use of introgression lines for QTL mapping and characterization in the maize - Setosphaeria turcica pathosystem
Source: BMC Plant Biol. 2010 Jun 8;10:103. doi: 10.1186/1471-2229-10-103 (PMC3017769; doi:10.1186/1471-2229-10-103)
Supplement: Additional file 1 — NLB resistance of the full set of 82 TBBC3 introgression lines. Relative area under the disease progress curve (Relative AUDPC) values shown are the differences of least squares means (from mixed models) between TBBC3 lines and B73 recurrent parent. AUDPC was calculated from three diseased leaf area (DLA) scores in the 2006 trial in NY (solid bars), or three disease severity scores in the 2006 trial in NC (open bars). In NY, primary DLA was also rated for diseased leaf area on inoculated leaves. The letters "R" and "S" below the graph indicate the lines significantly more resistant and more susceptible than B73 at P < 0.05, respectively, based on primary DLA and AUDPC. The 15 TBBC3 lines selected for subsequent phenotypic validation are indicated by rectangles highlighting the maize line designation. [file 1471-2229-10-103-S1.PDF]

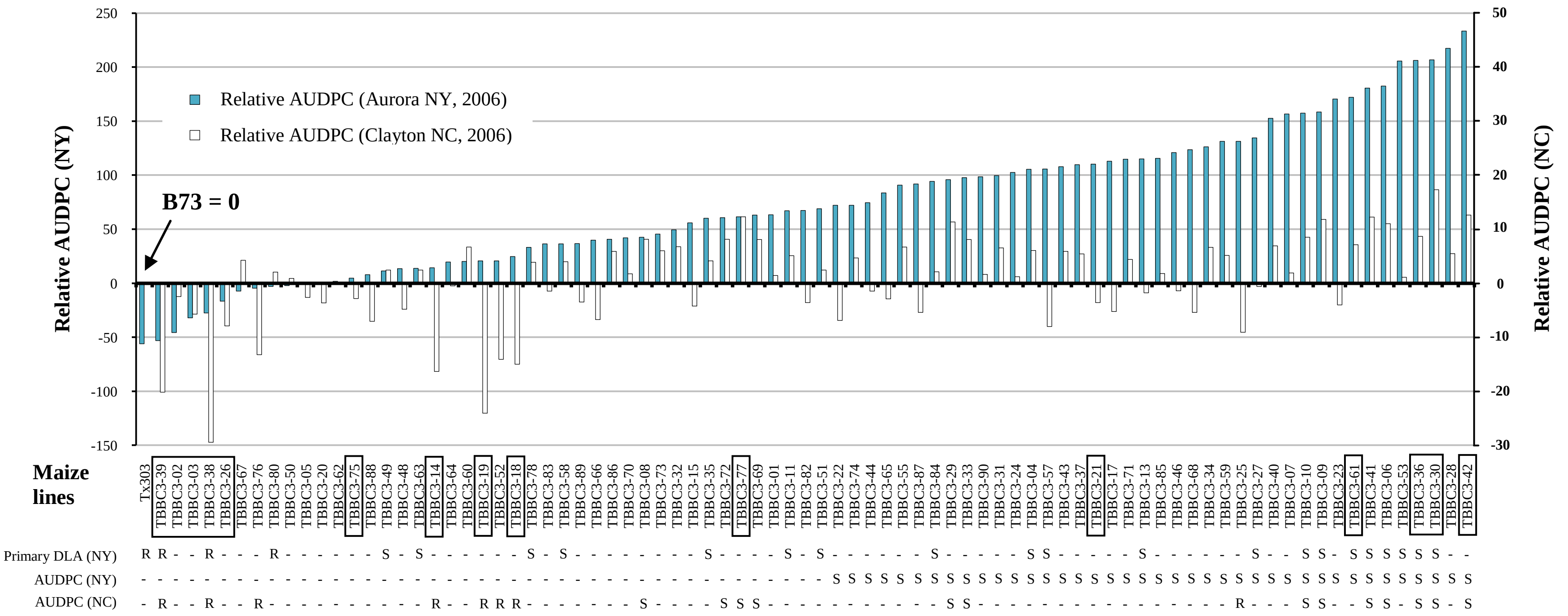

Additional file 1. NLB resistance of the full set of 82 TBBC3 introgression lines.

Relative area under the disease progress curve (Relative AUDPC) values shown are the differences of least squares means (from mixed models) between TBBC3 lines and B73 recurrent parent. AUDPC was calculated from three diseased leaf area (DLA) scores in the 2006 trial in NY (solid bars), or three disease severity scores in the 2006 trial in NC (open bars). In NY, primary DLA was also rated for diseased leaf area on inoculated leaves. The letters “R” and “S” below the graph indicate the lines significantly more resistant and more susceptible than B73 at $P < 0.05$, respectively, based on primary DLA and AUDPC. The 15 TBBC3 lines selected for subsequent phenotypic validation are indicated by rectangles highlighting the maize line designation.
