# Supplementary material for: Resistance loci affecting distinct stages of fungal pathogenesis: use of introgression lines for QTL mapping and characterization in the maize - Setosphaeria turcica pathosystem
Source: BMC Plant Biol. 2010 Jun 8;10:103. doi: 10.1186/1471-2229-10-103 (PMC3017769; doi:10.1186/1471-2229-10-103)
Supplement: Additional file 2 — Putative NLB QTL identified in the TBBC3 population. Putative QTL for northern leaf blight (NLB QTL) affecting incubation period (IP), primary diseased leaf area (PrimDLA), diseased leaf area (DLA), disease severity (severity), and AUDPC (area under the disease progress curve calculated from DLA or disease severity) were identified using 82 TBBC3 introgression lines. QTL effects for each marker locus are the significant differences of least squares means of Tx303 homozygous genotypes at the locus relative to B73 recurrent parent line (* 0.01 <P < 0.05, ** 0.001 <P < 0.01, *** P < 0.001). Putative QTL are reported as correlated groups because of the high dependencies among those introgressed segments in TBBC3 lines. [file 1471-2229-10-103-S2.PDF]

Additional file 2. Putative NLB QTL identified in the TBBC3 population.

Putative QTL for northern leaf blight (NLB QTL) affecting incubation period (IP), primary diseased leaf area (PrimDLA), diseased leaf area (DLA), disease severity (severity), and AUDPC (area under the disease progress curve calculated from DLA or disease severity) were identified using 82 TBBC3 introgression lines. QTL effects for each marker locus are the significant differences of least squares means of Tx303 homozygous genotypes at the locus relative to B73 recurrent parent line (* 0.01 < P < 0.05, ** 0.001 < P < 0.01, *** P < 0.001). Putative QTL are reported as correlated groups because of the high dependencies among those introgressed segments in TBBC3 lines.

Chr. Bin	Marker	Map Position [a]	Resistance allele	Aurora NY, 2006						Clayton NC, 2006				Previously reported NLB QTL at the locus	
				IP (days)	PrimDLA (%)	DLA1 (%)	DLA2 (%)	DLA3 (%)	AUDPC (%-day)	Severity1 (scale)	Severity2 (scale)	Severity3 (scale)	AUDPC (scale-day)	Mapping population [b]	Reference
Correlated loci															
1.01	*umc1071*	85.2	B73		6.6*		5.5***	8.0***	110.1***	0.7**				None	
1.02	*bnlg1429*	143.5	B73		6.9*	6.2***	7.1***	7.4**	134.7***					B52 x Mo17	[1]
	bnlg1953	170.0	B73		7.9**		6.3***	9.3***	120.0***	0.7**					
4.07	*bnlg1621*	349.6	B73		5.0*	3.0**	4.9***	9.4***	91.8***					D32 x D145	[2]
5.02	*bnlg565*	150.9	B73		4.6*	2.7***	4.9***	10.0***	90.7***					B52 x Mo17	[1, 3]
7.03	*bnlg434*	323.3	B73		6.6*	3.8***	4.7***	4.1*	84.0***					B52 x Mo17	[1, 3]
8.03 [d]	*UMC32B*	199.1	B73			8.8***	12.9***	23.5***	246.0***	1.3**			13.2**	None	
Correlated loci															
1.06 [c]	*umc2234*	529.0	Tx303							-1.1*			-12.2*	D32 x D145	[2]
5.08 [d]	*umc1225*	641.4	Tx303	1.6**	-18.6**					-2.0**	-1.9***	-1.2**	-31.9***	None	
	bnlg1695	664.2	Tx303	1.6**	-18.6**					-2.0**	-1.9***	-1.2**	-31.9***	None	
5.09 [d]	*umc1829*	671.5	Tx303	1.6**	-18.6**					-2.0**	-1.9***	-1.2**	-31.9***	None	
Correlated loci															
4.03	*umc2082*	141.6	B73		11.6***		7.4***	12.4***	132.9***	0.6*	0.4*		7.4*	D32 x D145	[2]
	umc2176	174.6	B73		11.6***		7.4***	12.4***	132.9***	0.6*	0.4*		7.4*		
5.04	*bnlg1208*	323.1	B73	-0.6*	15.4***	3.3**	6.3***	7.4**	103.8***	0.8*	0.5*	0.4*	10.2**	Lo951 x CML202	[4, 5]
														D32 x D145	[2]
Correlated loci															
1.11 [d]	*bnlg131*	1065.6	Tx303							-1.5***	-0.9*		-16.5**	IL731a x W6786	[6]
														Lo951 x CML202	[4, 5]
3.06 [d]	*bnlg2241*	452.7	Tx303							-1.5***	-0.9*		-16.5**	IL731a x W6786	[6]
														B52 x Mo17	[1]
														Lo951 x CML202	[4, 5]

[a] The marker position was based on genetic map of the intermated B73 x Mo17 population (version IBM 2008 neighbors).
[b] The mapping population in which the same locus was detected for resistance to NLB. The resistance donor was underlined.
[c] This marker was first found highly associated with NLB resistance in segregating populations B73 x TBBC3-38 and B73 x TBBC3-39. It was then tested in 82 TBBC3 lines.
[d] Introgressed segment at the locus was only present in a single TBBC line (bin 8.03 in TBBC3-42; bin 5.08-5.09 in TBBC3-38; bin 1.11 and 3.06 in TBBC3-30).

References

1. Freymark PJ, Lee M, Woodman WL, Martinson CA: **Quantitative and qualitative trait loci affecting host-plant response to *Exserohilum turcicum* in maize (*Zea mays* L.)**. *Theor. Appl. Genet.* 1993, **87**(5):537-544.
2. Welz HG, Xia XC, Bassetti P, Melchinger AE, Luebberstedt T: **QTLs for resistance to *Setosphaeria turcica* in an early maturing Dent x Flint maize population**. *Theor. Appl. Genet.* 1999, **99**(3-4):649-655.
3. Dingerdissen AL, Geiger HH, Lee M, Schechert A, Welz HG: **Interval mapping of genes for quantitative resistance of maize to *Setosphaeria turcica*, cause of northern leaf blight, in a tropical environment**. *Mol. Breed.* 1996, **2**(2):143-156.
4. Welz HG, Schechert AW, Geiger HH: **Dynamic gene action at QTLs for resistance to *Setosphaeria turcica* in maize**. *Theor. Appl. Genet.* 1999, **98**(6-7):1036-1045.
5. Schechert AW, Welz HG, Geiger HH: **QTL for resistance to *Setosphaeria turcica* in tropical African maize**. *Crop Sci.* 1999, **39**(2):514-523.
6. Brown AF, Juvik JA, Pataky JK: **Quantitative trait loci in sweet corn associated with partial resistance to Stewart's wilt, northern corn leaf blight, and common rust**. *Phytopathology* 2001, **91**(3):293-300.
