# Supplementary material for: Resistance loci affecting distinct stages of fungal pathogenesis: use of introgression lines for QTL mapping and characterization in the maize - Setosphaeria turcica pathosystem
Source: BMC Plant Biol. 2010 Jun 8;10:103. doi: 10.1186/1471-2229-10-103 (PMC3017769; doi:10.1186/1471-2229-10-103)
Supplement: Additional file 3 — Validation of NLB QTL in the BC4F2 segregating populations. The BC4F2 populations were genotyped and phenotyped for incubation period (IP), lesion expansion (LE), diseased leaf area (DLA), and area under the disease progress curve (AUDPC). The trait-marker association was tested by ANOVA at P < 0.05. Introgressions/markers significantly associated with NLB resistance are listed. The relative allele effects are the differences on the least squares means (LSMean) between Tx303 homozygous genotypes and B73 homozygous genotypes at the locus. [file 1471-2229-10-103-S3.PDF]

Additional file 3. Validation of NLB QTL in the BC_4F_2 segregating populations.

The BC_4F_2 populations were genotyped and phenotyped for incubation period (IP), lesion expansion (LE), diseased leaf area (DLA), and area under the disease progress curve (AUDPC). The trait-marker association was tested by ANOVA at $P < 0.05$. Introgressions/markers significantly associated with NLB resistance are listed. The relative allele effects are the differences on the least squares means (LSMean) between Tx303 homozygous genotypes and B73 homozygous genotypes at the locus.

Introgressed region (Chr. bin) [a]	Map interval (cM) [b]	SSR marker (map position, cM) [b]	Parental donor lines of the BC_4F_2 population [c]	Environment (location, year)	Resistance allele	R^2	Relative allele effect ($LSMean_{Tx303} - LSMean_{B73}$) [d]					
							IP (dpi)	LE (mm/day)	DLA1 (%)	DLA2 (%)	DLA3 (%)	AUDPC (%-day)
**1.06** [§]	487.5-590.5	*umc2234* (529.0)	TBBC3-38	Aurora NY, 2006	Tx303	0.330 [f]	1.96**	-0.29*	-6.36***	-6.69***	-5.31***	-119.27***
**1.06** [§]	487.5-590.5	*umc2234* (529.0)	TBBC3-39	Aurora NY, 2006	Tx303	0.526 [f]	2.38***	-0.33**	-5.79***	-8.39***	-9.54***	-151.70***
**1.01-1.02** [§]	0-184.2	*bnlg1953* (170.0)	TBBC3-42	Greenhouse, 2006	B73	0.139 [e]	-0.60***	0.35*	na	na	na	na
**1.01-1.02** [§]	0-184.2	*bnlg1953* (170.0)	TBBC3-77	Aurora NY, 2006	B73	0.074 [f]	-0.58	0.11	6.05***	3.36*	1.42	70.95*
5.00	0-30.0	*mmc0151* (20.5)	TBBC3-77	Aurora NY, 2006	B73	0.059 [f]	-0.71	0.26	4.30**	4.37**	3.04*	80.37**
5.01-5.02	115.1-153.9	*bnlg565* (150.9)	TBBC3-02	Aurora NY, 2006	B73	0.053 [e]	-1.30**	0.03	1.98	0.87	0.52	21.13
5.08-5.09	603.0-676.1	*bnlg1829* (313.3)	TBBC3-38	Aurora NY, 2006	Tx303	0.046 [f]	0.71	0.01	-2.02	-2.45*	-3.67*	-49.85**
8.03-8.05	265.2-344.0	*umc1130* (330.1)	TBBC3-02	Aurora NY, 2006	Tx303	0.114 [f]	0.41	-0.04	-2.97**	-3.90***	-3.02***	-68.90***

[§] The QTL effects on NLB resistance were further confirmed in derived BC_4F_3 and/or BC_4F_4 NILs.

[a] Introgressed regions that were significantly associated with traits in segregating populations. The genome regions are shown as bin positions (eg. bin 1.06 is the sixth segment in maize chromosome 1).

[b] SSR marker with the most significant effects on traits. The marker position was based on genetic map of the intermated B73 x Mo17 population (version IBM 2008 neighbors). The map interval of each introgression is assumed to extend halfway between two markers around the border of each end of the introgression.

[c] The selected TBBC3 lines used for developing the segregating BC_4F_2 populations. The lines were backcrossed to B73 and selfed. Phenotyping and genotyping were conducted on individual plants in the populations.

[d] The significance level was determined by pair-wise two-tailed Student's t test on the least squares difference (denoted as * $0.01 < P < 0.05$; ** $0.001 < P < 0.01$; *** $P < 0.001$).

[e] R-square from the ANOVA using IP as the dependent variable, referring to the proportion of the variation in IP accounted for by the marker in the third column.

[f] R-square from the ANOVA using AUDPC as the dependent variable, referring to the proportion of the variation in AUDPC accounted for by the marker in the third column.
