# Supplementary material for: Resistance loci affecting distinct stages of fungal pathogenesis: use of introgression lines for QTL mapping and characterization in the maize - Setosphaeria turcica pathosystem
Source: BMC Plant Biol. 2010 Jun 8;10:103. doi: 10.1186/1471-2229-10-103 (PMC3017769; doi:10.1186/1471-2229-10-103)
Supplement: Additional file 4 — Genotypes and disease phenotypes for Tx303, B73 and the NIL sets derived from B73 × TBBC3-38 and B73 × TBBC3-39. Among the target introgressions at bins 1.03, 1.06, 3.02, 5.00, 5.02-5.03 and 5.07-5.09, only qNLB1.06Tx303 (Tx303 allele at bin 1.06) was validated for association with resistance to NLB. The open bars and solid bars represent the loci homozygous for B73 alleles and Tx303 alleles, respectively. The gray bars represent heterozygous loci or missing genotypic data. Only the chromosomes with introgressed regions in the two NIL sets are shown. The rest of the genome was assumed fixed for B73 alleles. Trait values are least squares means calculated from the mixed model. Pair-wise Student's t tests were performed to analyze the differences between each NIL and B73, and between every pair of NILs in each set. Trait values with different letters are significantly different from each other. Disease phenotypes that were significantly more resistant than B73 are highlighted in bold and shaded, while the phenotypes significant more susceptible than B73 are underscored. Lines that showed significantly different days to anthesis are in bold italic. qNLB1.06Tx303 was also effective for resistance to Stewart's wilt. (IP: incubation period; LE: lesion expansion; DLA: diseased leaf area; PrimDLA: primary DLA; AUDPC: area under the disease progress curve) [file 1471-2229-10-103-S4.PDF]

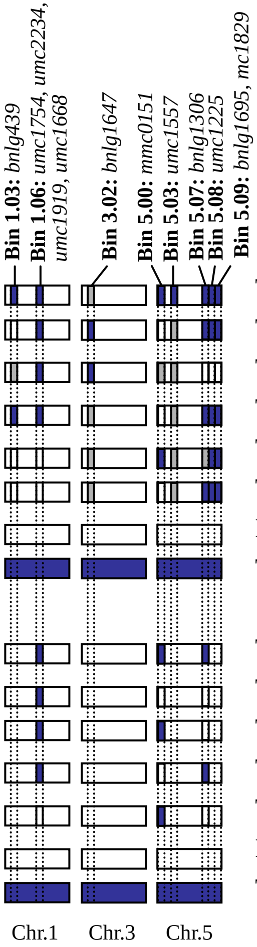

	Northern leaf blight							Stewart's wilt	Anthracnose stalk rot	Common smut		Common rust	Anthesis
	IP	LE	DLA1	DLA2	DLA3	DLA4	AUDPC	PrimDLA	Discolored internode area	Ear gall	Stalk gall	AUDPC	Days to anthesis
TBBC3-38	**17 A**	1.8 DEFG	**10 H**	**13 I**	**18 IJ**	**20 GH**	**462 J**	**0 N**	174 A	0 BC	0 B	53 H	***78 B***
TBBC3-38-11E	**15 ABC**	1.5 GH	15 DEFG	**14 HI**	**19 I**	**20 GH**	**501 IJ**	**16 JKL**				64 DEFGH	***77 BCDEFGH***
TBBC3-38-05F	**15 BCD**	1.4 H	**10 H**	**13 I**	**19 IJ**	**25 GH**	**504 IJ**	**16 KL**				70 DEFGH	75 EFGHI
TBBC3-38-18A	**14 CDEF**	1.6 FGH	**11 GH**	18 FGH	25 EFGH	**28 F**	**637 H**	**20 IJK**				55 GH	***78 BCD***
TBBC3-38-07H	**15 BCD**	1.6 FGH	13 DEFGH	22 DEFG	28 DEFG	38 CD	748 EFG	**30 HIJ**				59 FGH	***76 CDEFGH***
TBBC3-38-16G	13 DEFG	2.0 BCD	21 BC	28 C	29 DEF	34 DE	840 DE	48 DEF	141 CD	1.1 BC	0 B	74 DEF	***77 BC***
B73	13 F	1.5 H	15 DEF	21 EF	26 GH	33 E	710 G	44 F	142 D	0 C	0 B	61 GH	74 I
Tx303	**14 BCDE**	2.0 BCDE	12 EFGH	**14 HI**	**15 J**	**16 H**	**427 J**	**13 KLM**	**97 E**	2.5 A	1.6 A	83 CD	***87 A***
TBBC3-39	**15 BCD**	1.9 CDEF	12 FGH	**18 GHI**	**24 H**	**29 F**	**616 H**	**3 MN**	148 CD	0.1 BC	0 B	57 GH	***78 B***
TBBC3-39-19E	**16 AB**	1.6 FGH	13 EFGH	18 FG	**19 IJ**	**23 G**	**558 HI**	**5 LMN**	141 CD	0.1 BC	0 B	67 DEFGH	***75 FGH***
TBBC3-39-08F	**14 BCD**	1.6 FGH	13 DEFGH	**18 GHI**	25 FGH	**29 F**	**634 H**	**16 JKL**				61 FGH	***76 CDEFGH***
TBBC3-39-11A	12 FGHIJ	1.7 DEFGH	17 CD	26 CD	29 DE	35 CDE	813 DEF	**11 KLMN**				61 FGH	***76 CDEFGH***
TBBC3-39-09F	12 GHIJ	1.8 DEFG	16 DE	27 C	35 C	38 CD	885 D	**35 GH**	155 ABCD			64 EFGH	75 H
B73	13 F	1.5 H	15 DEF	21 EF	26 GH	33 E	710 G	44 F	142 D	0 C	0 B	61 GH	74 I
Tx303	**14 BCDE**	2.0 BCDE	12 EFGH	**14 HI**	**15 J**	**16 H**	**427 J**	**13 KLM**	**97 E**	2.5 A	1.6 A	83 CD	***87 A***

Additional file 4. Genotypes and disease phenotypes for Tx303, B73 and the NIL sets derived from B73 x TBBC3-38 and B73 x TBBC3-39.

Among the target introgressions at bins 1.03, 1.06, 3.02, 5.00, 5.02-5.03 and 5.07-5.09, only $qNLB1.06_{Tx303}$ (Tx303 allele at bin 1.06) was validated for association with resistance to NLB. The open bars and solid bars represent the loci homozygous for B73 alleles and Tx303 alleles, respectively. The gray bars represent heterozygous loci or missing genotypic data. Only the chromosomes with introgressed regions in the two NIL sets are shown. The rest of the genome was assumed fixed for B73 alleles. Trait values are least squares means calculated from the mixed model. Pair-wise Student's t tests were performed to analyze the differences between each NIL and B73, and between every pair of NILs in each set. Trait values with different letters are significantly different from each other. Disease phenotypes that were significantly more resistant than B73 are highlighted in bold and shaded, while the phenotypes significant more susceptible than B73 are underscored. Lines that showed significantly different days to anthesis are in bold italic. $qNLB1.06_{Tx303}$ was also effective for resistance to Stewart's wilt. (IP: incubation period; LE: lesion expansion; DLA: diseased leaf area; PrimDLA: primary DLA; AUDPC: area under the disease progress curve)
