# Supplementary material for: Resistance loci affecting distinct stages of fungal pathogenesis: use of introgression lines for QTL mapping and characterization in the maize - Setosphaeria turcica pathosystem
Source: BMC Plant Biol. 2010 Jun 8;10:103. doi: 10.1186/1471-2229-10-103 (PMC3017769; doi:10.1186/1471-2229-10-103)
Supplement: Additional file 5 — Genotypes and disease phenotypes for Tx303, B73 and the NIL set derived from B73 × TBBC3-42. Among all target introgressions at bins 1.01-1.02, 4.06-4.07, 5.02-5.03, 7.01, 8.02 and 8.03-8.05, only qNLB1.02B73 (B73 allele at bin 1.02) was validated for association with resistance to NLB. The open bars and solid bars represent the loci homozygous for B73 alleles and Tx303 alleles, respectively. The gray bars represent heterozygous loci or missing genotypic data. Only the chromosomes with introgressed regions in the NIL sets are shown. The rest of the genome was assumed fixed for B73 alleles. Trait values are least squares means calculated from the mixed model. Pair-wise Student's t tests were performed to analyze the differences between each NIL and B73, and between every pair of NILs. Trait values with different letters are significantly different from each other. Disease phenotypes that were significantly more resistant than B73 are highlighted in bold and shaded, while the phenotypes significant more susceptible than B73 are underscored. Lines that showed significantly different days to anthesis are in bold italic. Preliminary evidence also suggested that qNLB1.02B73 was effective for resistance to Stewart's wilt, and QTL at bin 1.01-1.02 and/or 5.02-5.03 were associated with resistance to common rust. [file 1471-2229-10-103-S5.PDF]

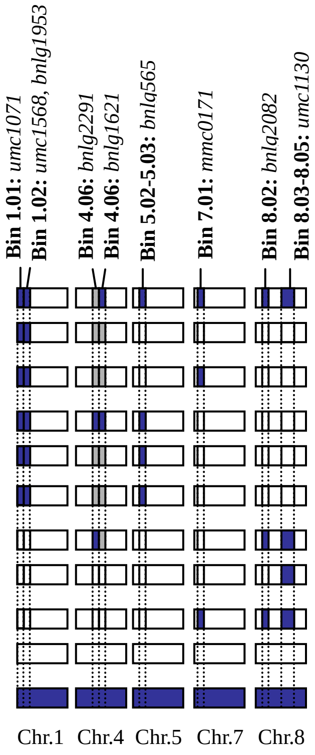

	Northern leaf blight							Stewart's wilt	Anthracnose stalk rot	Common smut		Common rust	Anthesis
	IP	LE	DLA1	DLA2	DLA3	DLA4	AUDPC	PrimDLA	Discolored internode area	Ear gall	Stalk gall	AUDPC	Days to anthesis
TBBC3-42	11 HIJ	2.5 A	30 A	45 A	57 A	67 A	1503 A	73 A	154 ABCD	0.5 BC	0 B	106 A	***76 CDEFG***
TBBC3-42-10E-02	11 HIJ	2.3 AB	33 A	46 A	55 A	71 A	1525 A	63 BC	139 CD			104 AB	***77 BCDE***
TBBC3-42-02E-01	11 IJ	2.2 ABC	31 A	45 A	56 A	71 A	1524 A	71 AB	167 ABC			115 A	***76 CDEFG***
TBBC3-42-06F-02	11 J	2.5 A	25 B	39 B	55 A	69 A	1406 B	58 CD	172 AB			84 BCDE	***76 CDEFGH***
TBBC3-42-12F-01	12 HIJ	2.1 ABC	25 B	38 B	49 B	60 B	1287 C	52 DE	148 ABCD			100 ABC	***76 CDEFGH***
TBBC3-42-10E-04										1.2 B	0.1 B	111 A	***77 BCDEF***
TBBC3-42-05H-04	12 GHIJ	1.5 GH	15 DEFG	24 CDE	30 D	40 C	814 DEF	48 DEF	145 BCD			65 DEFGH	***75 FGH***
TBBC3-42-05H-01	13 FGHI	1.5 FGH	15 DEFG	21 EFG	31 CD	37 CD	781 EF	**30 HI**	146 CD			74 DEFG	***76 DEFGH***
TBBC3-42-09E-01	13 EFGH	1.6 EFGH	15 DEFG	22 DEFG	27 DEFGH	36 CDE	743 FG	45 EFG	142 CD			65 EFGH	***75 GH***
B73	13 F	1.5 H	15 DEF	21 EF	26 GH	33 E	710 G	44 F	142 D	0 C	0 B	61 GH	74 I
Tx303	**14 BCDE**	2.0 BCDE	12 EFGH	**14 HI**	**15 J**	**16 H**	**427 J**	**13 KLM**	**97 E**	2.5 A	1.6 A	83 CD	***87 A***

Additional file 5. Genotypes and disease phenotypes for Tx303, B73 and the NIL set derived from B73 x TBBC3-42.

Among all target introgressions at bins 1.01-1.02, 4.06-4.07, 5.02-5.03, 7.01, 8.02 and 8.03-8.05, only $qNLB1.02_{B73}$ (B73 allele at bin 1.02) was validated for association with resistance to NLB. The open bars and solid bars represent the loci homozygous for B73 alleles and Tx303 alleles, respectively. The gray bars represent heterozygous loci or missing genotypic data. Only the chromosomes with introgressed regions in the NIL sets are shown. The rest of the genome was assumed fixed for B73 alleles. Trait values are least squares means calculated from the mixed model. Pair-wise Student's t tests were performed to analyze the differences between each NIL and B73, and between every pair of NILs. Trait values with different letters are significantly different from each other. Disease phenotypes that were significantly more resistant than B73 are highlighted in bold and shaded, while the phenotypes significant more susceptible than B73 are underscored. Lines that showed significantly different days to anthesis are in bold italic. Preliminary evidence also suggested that $qNLB1.02_{B73}$ was effective for resistance to Stewart's wilt, and QTL at bin 1.01-1.02 and/or 5.02-5.03 were associated with resistance to common rust. (IP: incubation period; LE: lesion expansion; DLA: diseased leaf area; PrimDLA: primary DLA; AUDPC: area under the disease progress curve)
